# Single Treatment of Mature 3D Single-, Dual- and Poly-Species Biofilms Using a Combination Therapy of Phage or Phage-Hetero-Cocktails and Ciprofloxacin

**DOI:** 10.3390/antibiotics15060537

**Published:** 2026-05-25

**Authors:** Tea Glonti, Merve Kübra Aktan, Christel Cochez, Naiera Zayed, Annabel Braem, Wim Teughels, Jean-Paul Pirnay

**Affiliations:** 1 Laboratory for Molecular and Cellular Technology, Queen Astrid Military Hospital, Bruynstraat 1, 1120 Brussels, Belgium; christel.cochez@mil.be (C.C.); jean-paul.pirnay@mil.be (J.-P.P.); 2 Biomaterials and Tissue Engineering Research Group, Department of Materials Engineering (MTM), KU Leuven, Kasteelpark Arenberg 44, 3001 Leuven, Belgium; mervekubra.aktan@kuleuven.be (M.K.A.); annabel.braem@kuleuven.be (A.B.); 3 Periodontology & Oral Microbiology, Oral Health Sciences, KU Leuven, Kapucijnenvoer 7, 3000 Leuven, Belgium; naiera.zayed@kuleuven.be (N.Z.); wim.teughels@kuleuven.be (W.T.)

**Keywords:** phage therapy, combination therapy, poly-biofilm, 3D biofilm, phage hetero-cocktails, phage-phage synergy, phage–antibiotic synergy, synergy level, persistence, re-sensitization

## Abstract

**Background/Objectives**: Biofilms are a form of defense that enables bacteria to withstand antibiotic pressure and demonstrate antibiotic resistance. It is crucial to develop anti-biofilm strategies in order to combat chronic and persistent multidrug-resistant (MDR) infections. **Methods**: In this study, we developed 3D biofilms of single-, dual-, and poly-species MDR ESKAPE components, including the pathogens *P. aeruginosa S. aureus* and *K. pneumoniae*, in CF Mu3Gel. We evaluated the efficacy of using a phage, a di-hetero phage cocktail or a poly-hetero phage cocktail in combination with ciprofloxacin to eliminate mature biofilm biomass after 72 h or one week in a single treatment. **Results**: The phage components mostly exhibited synergistic behavior when combined with ciprofloxacin and with each other in di- and poly-hetero-cocktails. The reduction in 72-h dual- and poly-species biofilms was one log higher than that of one-week biofilms treated with the phage–antibiotic combination. The greatest reductions were observed in the 72-h single-species biofilm with combination therapy, at 1.4–3.0 log. Reductions of 2.16 and 1.6 log were observed in the dual-species *P. aeruginosa* and *S. aureus* biofilm and the poly-species biofilm, respectively. **Conclusions**: This study examined how a single application of phages or phage cocktails, either alone or in combination with ciprofloxacin, impacted established biofilm models, and how this affected the proportion of microcolonies of different species within each model. These insights will facilitate the development of strategies for multiple follow-up treatments, as well as the reordering of phages, phage cocktails, and combinations with antibiotics, to improve outcomes. The 3D biofilm models developed here could be used to screen phages or phage cocktails either on their own or alongside other therapies. This would facilitate the application of in vitro findings to real physiological settings.

## 1. Introduction

Antibiotic resistance poses a significant challenge to global public health. One of the most significant factors contributing to the emergence of antibiotic resistance is the ability of biofilms to increase tolerance to antimicrobial agents. Biofilms increase bacteria’s resistance to a wide range of environmental factors, making them difficult to treat with antimicrobials. Up to 80% of clinical bacterial infections are associated with biofilms [1,2]. Bacteria embedded in biofilms can be 1000 times more resistant to antimicrobials than planktonic bacteria [3]. Biofilm formation is associated with most chronic human infections [4,5] and is crucial for the long-term survival of pathogens. Natural biofilms in the environment and in hosts are often multispecies communities that differ greatly from mono-species biofilm systems in composition, structure, and antimicrobial resistance [6,7].

Polymicrobial infections involving *Pseudomonas aeruginosa*, *Staphylococcus aureus,* and *Klebsiella pneumoniae* are frequently reported in clinical settings [5], including in patients with cystic fibrosis (CF) [7,8,9,10], urinary tract infections, and wound infections [11,12]. These bacteria exhibit strong virulence factors and the ability to produce biofilms, enabling them to initiate infectious diseases [13]. These infections are characterized by poor clinical outcomes and increased antibiotic resistance [12,14]. The dynamics of lung colonization are defined by the co-colonization of *P. aeruginosa* by different species, including *S. aureus* and *K. pneumoniae*, and their interaction through competition and/or cooperation [15]. This results in a longer duration and more complicated infection [5,16]. Therefore, studying prevalent combinations of bacterial infections is important yet challenging. Importantly, *P. aeruginosa*, *S. aureus*, and *K. pneumoniae* are multidrug-resistant (MDR) ESKAPE pathogens. Carbapenem-resistant *K. pneumoniae* is classified as a critical priority pathogen [17]. Meanwhile, carbapenem-resistant *P. aeruginosa* and methicillin-resistant *S. aureus* are higher-priority pathogens on the 2024 World Health Organization [18] list of priority bacterial pathogens. These pathogens pose a significant threat to public health due to the limited treatment options available.

MDR pathogens are a cause for concern and necessitate the development of unconventional therapies. Among these therapies, phage therapy is a promising treatment strategy [17,19], as evidenced by its successful use in recent compassionate cases in Europe and the United States. Phages have several advantages over antibiotics. They typically target only one species or strain of bacteria, leaving the host’s commensal microbiota largely unharmed. Phages can effectively lyse MDR pathogens. They replicate at the active infection site, providing a sustained therapeutic effect [20]. Since the 1990s, phages have been considered a potential treatment for multidrug-resistant (MDR) diseases [21]. Several studies have demonstrated the effectiveness of using phages to treat MDR bacterial infections in vitro [22,23,24,25]. Recently, Pirnay et al. published an analysis of successful clinical outcomes with personalized phages. They reported clinical improvement and eradication of the targeted bacteria in 77.2% and 61.3% of infections, respectively. In vivo bacteriophage resistance selection and in vitro phage–antibiotic synergy were documented in 43.8% and 90% of the evaluated patients, respectively [26]. Schooley et al. published significant findings regarding the efficacy and health benefits of personalized phage treatment [27].

Phages can be used alongside standard antibiotics without affecting their antibacterial pharmacodynamic activity. The phage–antibiotic combination is a promising complementary therapy for treating MDR bacterial infections [19,28,29,30]. Gharieb et al. demonstrated the anti-biofilm potential of lytic phages against MDR *S. aureus* biofilms [31]. Bacteriophages can infect metabolically inactive persister cells embedded in biofilms, which are highly resistant to antibiotics [32]. Developing potential strategies to combat persister cells is highly significant from a clinical standpoint [33]. A combination of phages and antibiotics has been shown to significantly reduce the biofilms of different pathogens [34,35,36,37,38,39,40,41].

Phage cocktails are an encouraging approach for treating MDR infections and biofilms. Forti et al. treated biofilms with a phage cocktail, demonstrating that the phages could reach and reduce the biomass of bacteria embedded within the biofilms. Furthermore, they demonstrated the potential of phage cocktail therapy by effectively reducing infections in mice and treating bacteriemia in wax moths (*Galeria mellonella*) [20]. Several studies have demonstrated the successful use of phage cocktails in treating biofilms [42,43].

In order to study biofilm structure and formation, exopolysaccharide (EPS) production, metabolic activity, microbial behavior, and antibiotic resistance, as well as to screen different antimicrobials and translate their effects in vivo [4], it is important to develop a biofilm pattern that mimics the physiologically relevant conditions of the host. The main models of bacterial biofilms include in vitro static models (e.g., microtiter plates), dynamic systems (e.g., flow cells, continuous-flow reactors, 3D biofilms, and microcosms), specialized models, and ex vivo models [21,44]. Pacheco et al. developed and tested mucin-containing hydrogels (CF-Mu3Gel), which are dynamic, heterogeneous, three-dimensional (3D) structures that mimic the human body’s physiological setting. These structures include crosslinking and oxygen gradients. Specifically, the CF-Mu3Gel mimics the pattern of cystic fibrosis (CF) airway mucus, facilitating the study of interactions between different bacterial species [45].

In this study, we developed single-, dual- and poly-species 3D biofilms of *P. aeruginosa*, *S. aureus* and *K. pneumoniae* in CF Mu3Gel. These biofilms matured over periods of 72 h and one week. Our goals were threefold: first, to evaluate the efficacy of using a phage, a phage di-hetero-cocktail, or a phage poly-hetero-cocktail in combination with an antibiotic for the one-shot elimination of mature biofilm biomass; second, to demonstrate the synergy between the phages in the hetero-cocktail and the antibiotic; and third, to demonstrate how phages can modify the composition of biofilms, which could counteract the persistent and resilient nature of biofilm-associated pathogens.

We studied the relative abundance of different species in biofilms of combined models, both with and without exposure to an antibiotic, a phage, or a phage cocktail, either alone or in combination. Consequently, we performed a comparative analysis of the results from the different biofilm models. Additionally, we evaluated the synergistic interactions of each component and their persistence in dual- and poly-species biofilms. We also examined how the proportion of persistence of the different species changes under antimicrobial exposure. The results showed that *P. aeruginosa* can become sensitive to ciprofloxacin again when treated with a combination of phage cocktails and antibiotics. These findings provide insight into how the proportion of different species in microcolonies changes in each biofilm model. These findings will help further develop strategies for multiple follow-up treatments and reordering phage, phage cocktail, and antibiotic combinations to improve outcomes.

In the future, these 3D biofilm models in CF Mu3Gel could be used to screen phages or phage cocktails, either alone or in combination with other therapies. This would facilitate the translating of in vitro experience into real physiological settings, and would be useful for studying the interaction between different species, as well as variations within the same species, in settings that are as similar to nature as possible.

## 2. Results

### 2.1. Phage Lytic Activity on Planktonic Cultures and MIC

The *S. aureus* ATCC 6538, *P. aeruginosa* PAO1K and *K. pneumoniae* ATCC 27736 bacterial strains used in this study had previously been tested with phages and their susceptibility to the *S. aureus* phage Sb1, the *P. aeruginosa* phage Atpa010 and the *K. pneumoniae* phage Atkp010 had been determined. The *P. aeruginosa* phage Atpa010 and the *K. pneumoniae* phage Atkp010 phages were previously screened on 102 *P. aeruginosa* and 155 *K. pneumoniae* strains with different genetic backgrounds and capsular types, and their lytic activity was investigated [46]. The *S. aureus* phage Sb1 was known from previous studies [47]. The phage Sb1, from the George Eliava Institute of Bacteriophages, Microbiology and Virology, is well known for its high lytic activity. All three phages cleared the respective planktonic cultures (with an initial inoculum of 5 × 10^5^ CFU/mL, low-load planktonic cultures (LLPC)) at a ratio of 0.1 phages to bacteria for 48 h (Figure S1).

**Figure 1:**
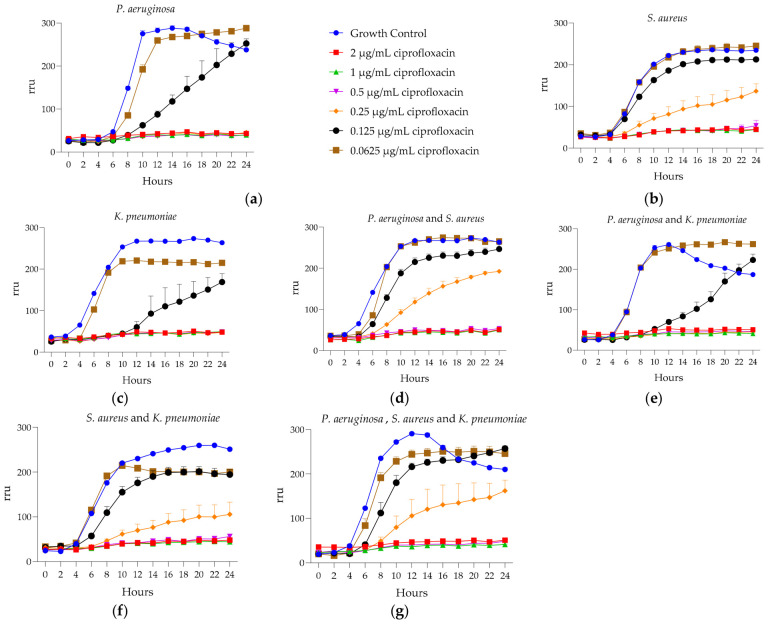
The proliferation curves of the low-load planktonic cultures of (**a**) *P. aeruginosa*, (**b**) *S. aureus* (**c**) *K. pneumoniae*, (**d**) *P. aeruginosa* and *S. aureus*, (**e**) *P. aeruginosa* and *K. pneumoniae*, (**f**) *S. aureus* and *K. pneumoniae*, and (**g**) *P. aeruginosa*, *S. aureus*, and *K. pneumoniae* exposed to different concentrations of ciprofloxacin (2 µg/mL − 0.0625 µg/mL). The (**a**) legend applies to all tetrazolium reduction curves. The tetrazolium reduction is expressed in relative respiration units (RRUs). Values are the mean ± SD of triplicates (*n* = 3).

Ciprofloxacin was selected based on a preliminary study because there was little difference in the Minimum Inhibitory Concentrations (MIC) of ciprofloxacin for all three strains compared to those of other antibiotics (Colistin and Meropenem). We calculated the MICs of a low-load planktonic culture (EUCAST standard of 5 × 10^5^ CFU/mL) in the OmniLog system, which generates high-throughput kinetic readouts based on bacterial cell metabolic activity. This allowed us to directly compare the differences and assess the effect of ciprofloxacin on bacterial growth [46] (Figure 1). The standard MIC determination tests for all three pathogens against the different antibiotics were performed using the VITEK 2 (Ref. 27226, BioMérieux, Inc., Salt Lake City, UT, USA) at the QAMH Military Medical Laboratory Capacity (MMLC) ISO 15189 (Figure S2). The MIC for *S. aureus* ATCC 6538, *P. aeruginosa* PAO1K and *K. pneumoniae* ATCC 27736 against ciprofloxacin were 0.5 µg/mL, 0.25 µg/mL and 0.125 µg/mL, respectively. Regarding co-culturing, the MICs of dual-species planktonic cultures of *P. aeruginosa* and *S. aureus*, *P. aeruginosa* and *K. pneumoniae*, and *S. aureus* and *K. pneumoniae*, as well as poly-species planktonic cultures, were all 0.5 µg/mL, the same as the MIC of a single planktonic culture of *S. aureus*, which was the highest of the three (Figure 1).

However, to determine the appropriate dosage of ciprofloxacin for treating mature biofilms with a bacterial load ranging from 1.0 × 10^8^ to 1.0 × 10^9^ CFU/mL (different from the EUCAST standard of 5.0 × 10^5^ CFU/mL), we performed an MIC test against ciprofloxacin with high-load planktonic cultures (HLPC) (final concentration of 5.0 × 10^8^, high-load planktonic culture) of the aforementioned combinations. We considered this approach to determine the minimum bactericidal biofilm concentration (MBBC) [48]. The results were calculated based on Relative Respiration Unit (RRU) values (Figure S3). The results differ greatly from those of the MIC against ciprofloxacin of a low-load planktonic culture.

**Figure 2:**
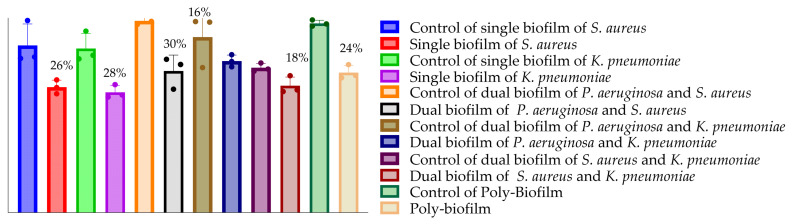
Percent reduction of high-load planktonic cultures and co-cultures at 1–8 µg/mL of ciprofloxacin. Values are the mean ± SD of triplicates (*n* = 3).

Here, we could only evaluate the MBBC for *P. aeruginosa*, which is 2 µg/mL. This is higher than the 0.5 µg/mL observed in the low-load planktonic culture. However, the RRU reduction at 1–8 µg/mL of ciprofloxacin is nearly identical in the proliferation curves of all the other cultures, as shown in Figure S3. Figure 2 shows the percentage reduction in ciprofloxacin at concentrations ranging from 1 to 8 µg/mL, calculated based on RRU reduction values. (Figure 2, Table S1).

Based on these results and data from the publication [6], we selected 4 µg/mL of ciprofloxacin to treat different biofilm models, both alone and in combination with phage hetero cocktails that would not interfere with the phages or phage cocktails. This enabled us to evaluate phage activity while treating the test biofilms. Regarding phage lytic activity, a ratio of 100 phages to bacteria resulted in clearance for only 12 h when high-load planktonic cultures were used (Figure S4). Using the interpretation model that we previously developed, we can analyze Figure S4 and observe the bacterial inhibition effect by phage [46]. The bacteria–phage proliferation curves of all cases have shorter and longer exponential phases and express reduced and almost constant RRU, which is maintained up to 24 h. We did not observe this type of proliferation curve in low-load planktonic cultures (Figure S1), even though the MOI of phages to bacteria was the same. This refers to the slow growth of bacterial cells, including phage-resistant mutants under a high planktonic culture load. Thus, a ratio of 100 phages to bacteria was used to treat all biofilm models. This approach was suggested in another publication [49].

### 2.2. Biofilm Formation

Single-, dual-, and poly-species biofilms were developed and examined using two models: a 96-well plate and a microscopic glass slide, both containing CF Mu3Gel. Both models were tested for biofilm formation over 72 h and one week. The 96-well plates with CF Mu3Gel were primarily used for analysis in the OMNILOG system and for enumeration of CFU/mL. Biofilm models were established on microscopic glass slides using CF Mu3Gel for scanning electron microscopy (SEM), confocal laser scanning microscopy (CLSM), and enumeration of CFU/mL.

#### 2.2.1. Enumeration of the Bacterial Load in Biofilm Models

Preliminary experiments were conducted to standardize the enumeration assay of bacterial loads (CFU/mL) for the biofilm models of both conditions, in both 96-well plates and on microscopic glass slides, to ensure comparability with each other and with the results of SEM and CLSM images.

**Figure 3:**
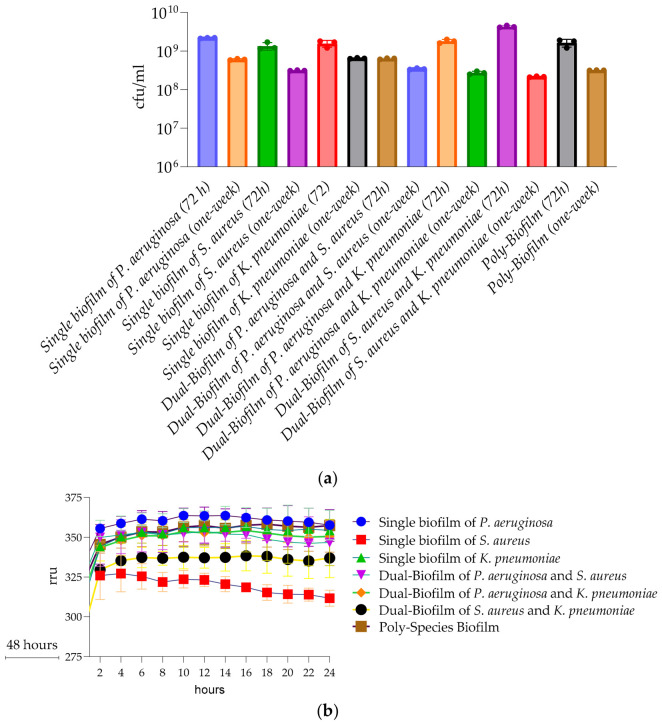
Bacterial load in 72-h biofilm models: (**a**) Bacterial load expressed as CFU/mL. (**b**) Bacterial load is expressed as Relative Respiratory Units (RRUs) in the OMNILOG system; the tetrazolium dye was added after 48 h, and the OmniLog system was used to read the results for the last 24 h. Values are the mean ± SD of triplicates (*n* = 3).

Figure 3a illustrates the biofilm load in CFU/mL over 72 h and one-week. After 72 h, the bacterial load in single biofilms of *P. aeruginosa S. aureus*, and *K. pneumoniae,* and dual-species biofilms of *P. aeruginosa* and *S. aureus*, *P. aeruginosa* and *K. pneumoniae*, and *S. aureus* and *K. pneumoniae*, as well as in poly-species biofilms, reached 2.17 × 10^9^ CFU/mL, 1.16 × 10^9^ CFU/mL, 1.18 × 10^9^ CFU/mL, 6.34 × 10^8^ CFU/mL, 1.78 × 10^9^ CFU/mL, 4.40 × 10^9^ CFU/mL and 1.92 × 10^9^ CFU/mL, respectively. However, after one week, the biomass of each model decreased by 3.55 × 10^0^ CFU/mL, 3.69 × 10^0^ CFU/mL and 1.81 × 10^0^ CFU/mL, 6.35 × 10^0^ CFU/mL, 2.06 × 10^0^ CFU/mL and 6.03 × 10^0^ CFU/mL (Table 1). Figure 3b shows the reduction of a tetrazolium dye in RRU of 72-h biofilms in the OMNILOG system, with values ranging from 312 to 360 RRU. The RRU expressions for one-week biofilms were almost the same as for 72-h biofilms, as only the last 24 h were read.

#### 2.2.2. Scanning Electron Microscopy (SEM)

**Figure 4:**
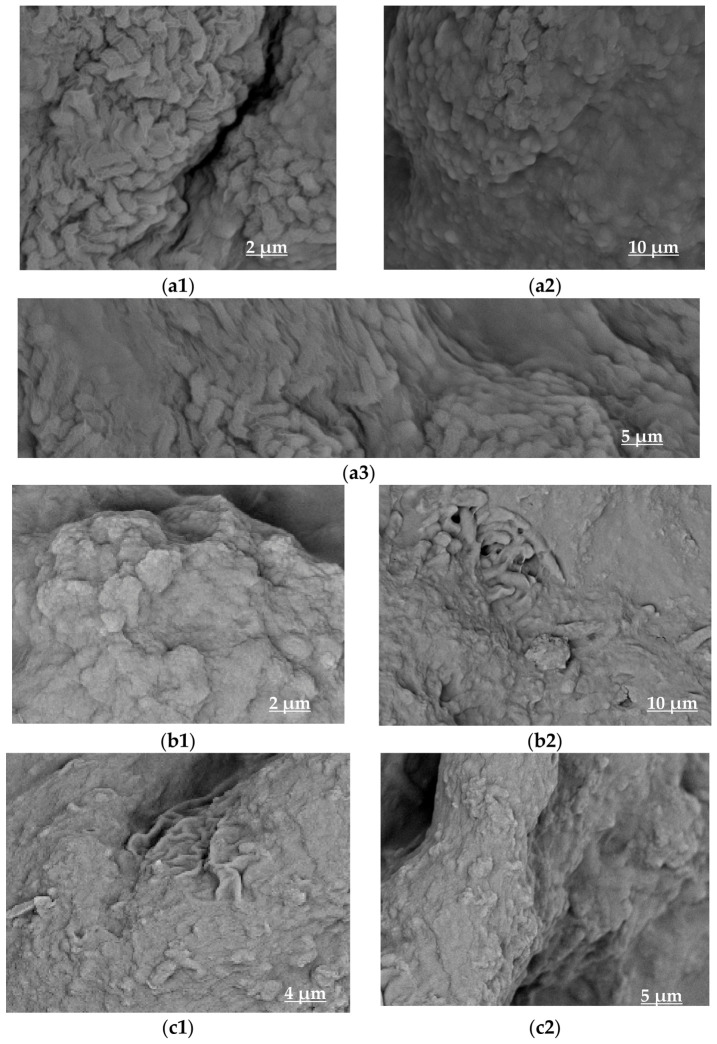
SEM pictures of mature biofilms after one week: (**a1**–**a3**) are images of poly-species biofilms of different zones and dimensions; (**b1**,**b2**) are images of dual-species biofilms of *P. aeruginosa* and *S. aureus* of different zones and dimensions; and (**c1**,**c2**) are images of dual-species biofilm of *P. aeruginosa* and *K. pneumoniae* of different zones and dimensions.

We observed the structural architecture of one-week dual-species (*P. aeruginosa* and *S. aureus*, *P. aeruginosa* and *K. pneumoniae*, *S. aureus* and *K. pneumoniae*) and poly-species (*P. aeruginosa*, *S. aureus*, and *K. pneumoniae*) biofilms using scanning electron microscopy (SEM). SEM images (Figure 4) show mature biofilms after one week, with a slimy and intensive protective matrix of EPS embedded within the pathogens at high density. Furthermore, the combination of different species in biofilm models results in different structures: the poly-species biofilm (Figure 4(a1–a3)) is characterized by a smooth layer (shell) covering cell aggregates; also, this kind of channel (Figure 4(b2)) could be a broken-off biofilm, indicating the dispersal of bacterial cells to repopulate another area. The dual-species biofilm of *P. aeruginosa* and *S. aureus* reveals a hill showing a mushroom-like appearance (Figure 4(b1)) and valley [50] structure of biofilm, with large aggregates of dense pathogens embedded (Figure 4(c2)). Meanwhile, the dual-species biofilm of *S. aureus* and *K. pneumoniae* is characterized by wrinkled shell covering cell aggregates (Figure 4(c1)).

#### 2.2.3. Confocal Laser Scanning Microscopy (CLSM)

**Figure 5:**
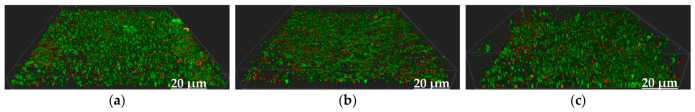
Different captures of CLSM images of one-week poly-biofilms of *P. aeruginosa*, *S. aureus*, and *K. pneumoniae*: (**a**–**c**). The biofilms were stained using the LIVE/DEAD™ BacLight™ Bacterial Viability Kit. Live cells stain green and dead cells stain red.

Only the one-week poly-biofilm was studied under CLSM. Figure 5 shows 3D structures, biomass composition (live and dead cells), and biofilm dynamics in real time. The matrixes consist of spatially distributed microcolonies that are interconnected.

#### 2.2.4. Determining the Load of Each Bacterial Strain in 72-h and One-Week Biofilm Models

**Figure 6:**
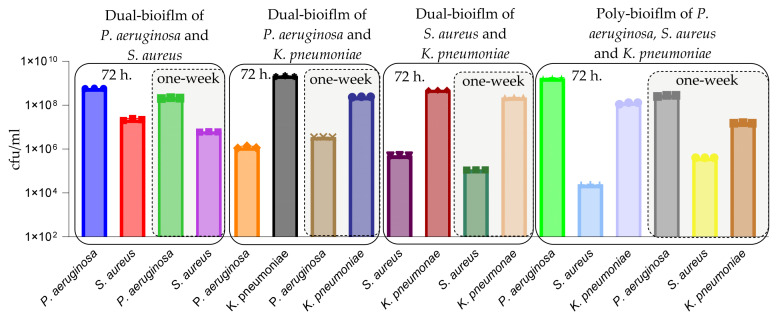
The load of bacterial strains in untreated dual- and poly-species biofilm models after 72 h and one week. The values are the mean ± SD of triplicates (*n* = 3).

Figure 6 demonstrate the load of each bacterial strain in dual and poly-biofilms. There are slight differences between the 72-h and one-week biofilms. The ratio of each bacterial strain was calculated using the standard division formula in Excel. In untreated dual-species biofilms, *P. aeruginosa* dominates *S. aureus* with 2.62 × 10^1^ and 5.66 × 10^1^, respectively. In triple-species biofilms, *P. aeruginosa* dominates both *S. aureus* with 1.03 × 10^5^ and 6.75 × 10^2^, respectively, and *K. pneumoniae* with 1.95 × 10^1^ and 1.76 × 10^1^, respectively. Meanwhile, *K. pneumoniae* dominates *S. aureus* with 8.16 × 10^1^ and 2.03 × 10^3^, respectively, and *P. aeruginosa* with 1.38 × 10^3^ and 6.70 × 10^1^, respectively, in dual-species biofilms.

### 2.3. Treatment of Biofilm Models

We treated all 72-h and one-week matured biofilm models in a single round for 24 h. The formulations of phages, phage cocktails, and ciprofloxacin, both alone and in combination, in 100 µL and 200 µL suspensions, which were applied to treat biofilms in one well of a 96-well plate and on a microscopic glass slide, are presented in the Section 4.

The type of interaction between the phage or phage cocktail and antibiotic that produced a more potent effect over the course of the experiment than the single agents was classified as synergistic activity [46]. The type of interaction between the phage or phage cocktail and antibiotic that produced the same potent effect as the single agents was classified as proto-cooperation [46].

#### 2.3.1. Results of the Enumeration of Bacterial Strains in the Treated Biofilm Models

**Figure 7:**
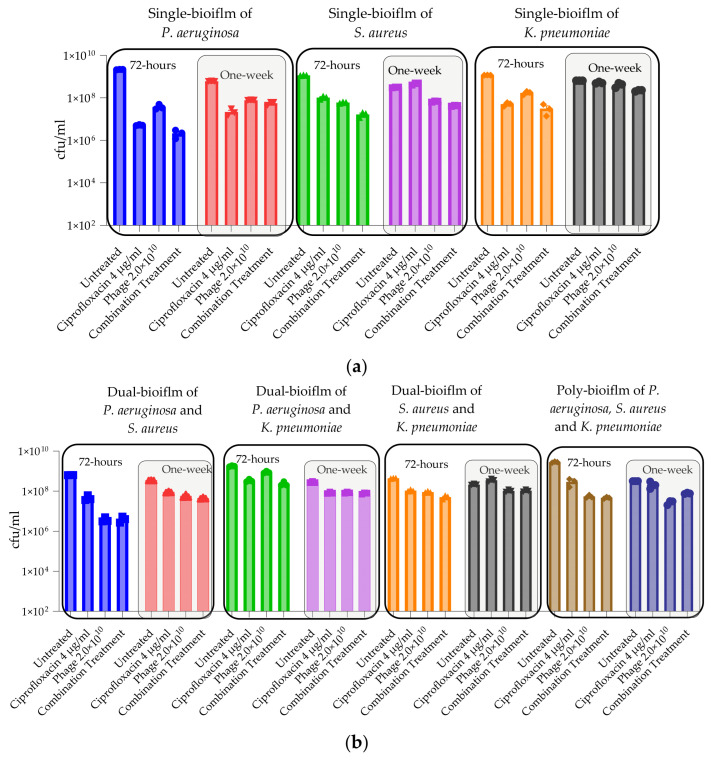
Results of a single 24-h treatment for (**a**) 72-h-old and (**b**) one-week-old single-, dual-. and poly-species biofilms. The values are the mean ± SD of triplicates (*n* = 3).

The greatest reductions in total biomass with combination therapy were observed in the 72-h single-species biofilms of *P. aeruginosa, S. aureus*, and *K. pneumoniae*, followed by the dual-species biofilm of *P. aeruginosa* and *S. aureus*, and then the poly-species biofilm. Relatively lower reductions were observed in the 72-h dual-species biofilms of *P. aeruginosa* and *K. pneumoniae*, as well as *S. aureus* and *K. pneumoniae* (Figure 7).

The greatest reductions by the phages and phage cocktails in one-week biofilms were observed in the poly-species biofilm and the single-species biofilm of *P. aeruginosa*. We found that the *K. pneumoniae* phage alone had more difficulty treating biofilm-associated bacterial cells than the *P. aeruginosa* and *S. aureus* phages, exhibiting a maximum reduction of 0.7 log in the 72-h single biofilm (Figure 7).

#### 2.3.2. Interpretation of Phage and Ciprofloxacin Interaction

In this study, we developed criteria for evaluating the level of synergy. A reduction of 0.7 log or more in the combination of a phage or a phage cocktail with ciprofloxacin was considered to be a high level of synergy; a reduction of 0.3–0.5 log in the combination was considered to be a medium level of synergy; a reduction of 0.15–0.25 log in the combination was considered to be a low level of synergy; and a reduction of 0.08–0.1 was considered to be negligible (Table 2). Additionally, overgrowth of bacteria under antibiotic exposure alone was evaluated as a negative effect.

A high degree of synergy was exhibited by phages and ciprofloxacin in 72-h single-species biofilms of *P. aeruginosa* and *S. aureus*. However, in the other biofilm models, the synergy was low, except for the dual-species biofilm of *P. aeruginosa* and *S. aureus*, where proto-cooperation was established. In particular, a low degree of synergistic activity was revealed in dual-species biofilms of *P. aeruginosa* and *S. aureus*, and a medium degree was revealed in the single-species *S. aureus* biofilm. This last case is interesting because the synergistic effect is due to phage activity, whereas ciprofloxacin caused a negative effect, resulting in an increase in the number of *S. aureus* colonies compared to the control (Table 2). This could be an example of *S. aureus* hypothesizing that phages remain active against antibiotic-tolerant and less active bacteria, while most phage population growth involves metabolically active bacterial cells [51].

#### 2.3.3. Overall Interpretation of the Results of the Biofilm Model Treatment

The overall interpretation of the results of the single-, dual-, and poly-species biofilm treatments is provided in Table 3.

#### 2.3.4. Determining the Proportions of Bacterial Strains in 72-h and One-Week-Old Biofilm Models Following a Single 24-h Treatment

**Figure 8:**
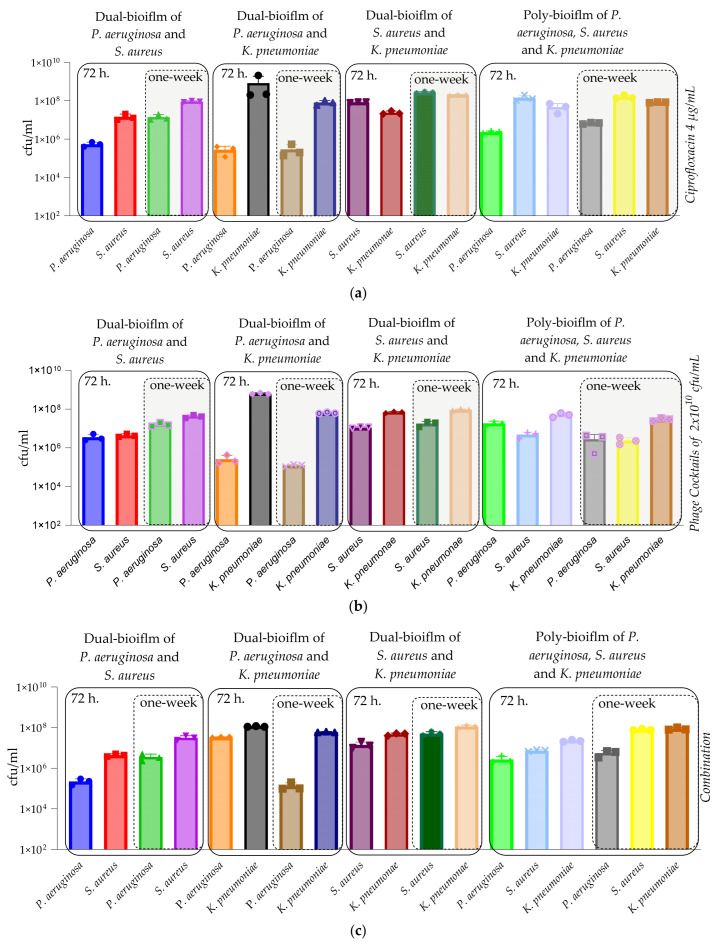
The proportions of bacterial strains in 72-h and one-week-old dual- and poly-species biofilm models after 24 hours’ exposure to: (**a**) ciprofloxacin alone, (**b**) a phage cocktail alone, or (**c**) combination. Values are means ± SD of triplicates (*n* = 3).

Figure 8 shows the results of the enumeration (CFU/mL) of the individual strains and their proportion in the treated biofilm models. As can be seen, *S. aureus* and *K. pneumoniae* show greater resistance than *P. aeruginosa*. In dual- and poly-species biofilms exposed to ciprofloxacin, *S. aureus* dominates *P. aeruginosa* by 0.7–1.6 log CFU/mL and slightly dominates *K. pneumoniae*. Furthermore, *P. aeruginosa* and *S. aureus* phages demonstrate good efficacy, both alone and in combination. In contrast, *K. pneumoniae* phage exhibits significantly lower efficacy. In biofilm models under phage cocktail exposure, *K. pneumoniae* dominates the other two strains, particularly *P. aeruginosa*.

#### 2.3.5. The Results of Tetrazolium Reduction in the OMNILOG System

**Figure 9:**
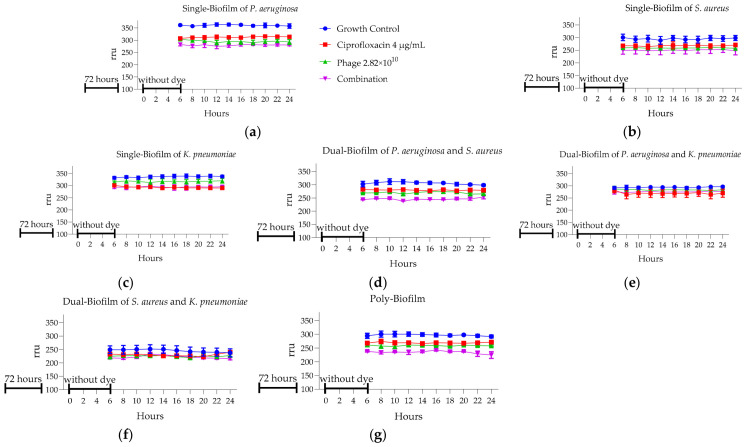
The tetrazolium reduction curves of the single-species biofilms of (**a**) *P. aeruginosa*, (**b**) *S. aureus*, (**c**) *K. pneumoniae*, of dual-species biofilms of (**d**) *P. aeruginosa* and *S. aureus*, (**e**) *P. aeruginosa* and *K. pneumoniae*, (**f**) *S. aureus* and *K. pneumoniae*, and of poly-species biofilm of (**g**) *P. aeruginosa*, *S. aureus*, and *K. pneumoniae* exposed to ciprofloxacin and phage or phage cocktail, alone and in combination. The (**a**) legend applies to all reduction curves. Values are means ± SD of triplicates (*n* = 3).

We tried to observe biofilm model proliferation curves in the OmniLog system. However, seeing a reduction curve is very unlikely with mature biofilms, because the high cell density leads to rapid saturation of the reaction. This limits the assay’s sensitivity and dynamic range. For this reason, we added dye to the mature biofilms after six hours of exposure to antimicrobials, and then continued their incubation in the OmniLog System for a further 18 h. The proliferation curve of single-species biofilms of *P. aeruginosa* and *S. aureus* is reduced by 100 RRU. Dual-species biofilms of *P. aeruginosa* and *S. aureus*, as well as poly-species biofilms’ proliferation curves, are reduced by 150 RRU. All other biofilms’ proliferation curves are reduced by 50 RRU (Figure 9). We suppose that the tetrazolium reduction readings were based on the results of surface-grown pathogen populations. Bacteria located on metabolically active biofilm surfaces could be primary targets for phage replication and antibiotics [51].

#### 2.3.6. Determination of Phage Titer (PFU/mL) in Tested Biofilm Models

**Figure 10:**
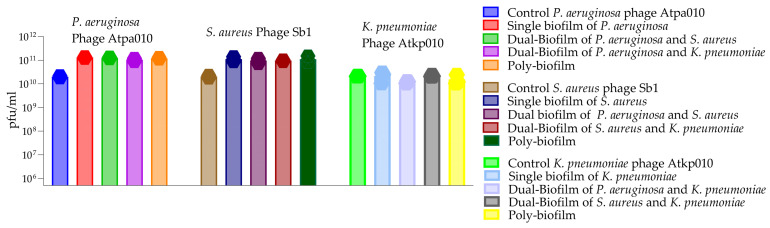
Results of phage titration in treated biofilms; values are the mean of triplicates (*n* = 3).

The same sample dilutions that were used to enumerate the bacterial load were also used to enumerate the phage particles (PFU/mL). We observed an increase in phage titers after treating mature biofilms for 24 h. The *P. aeruginosa* phage Atpa010 showed a 0.6–0.7 log increase, and the *S. aureus* phage Sb1 showed a 0.5 log increase. There was no notable increase in *K. pneumoniae* phage Atkp010. Its titers remained consistent across all biofilm models (Figure 10).

#### 2.3.7. SEM Visualization of the Effect of Treating the Biofilm

**Figure 11:**
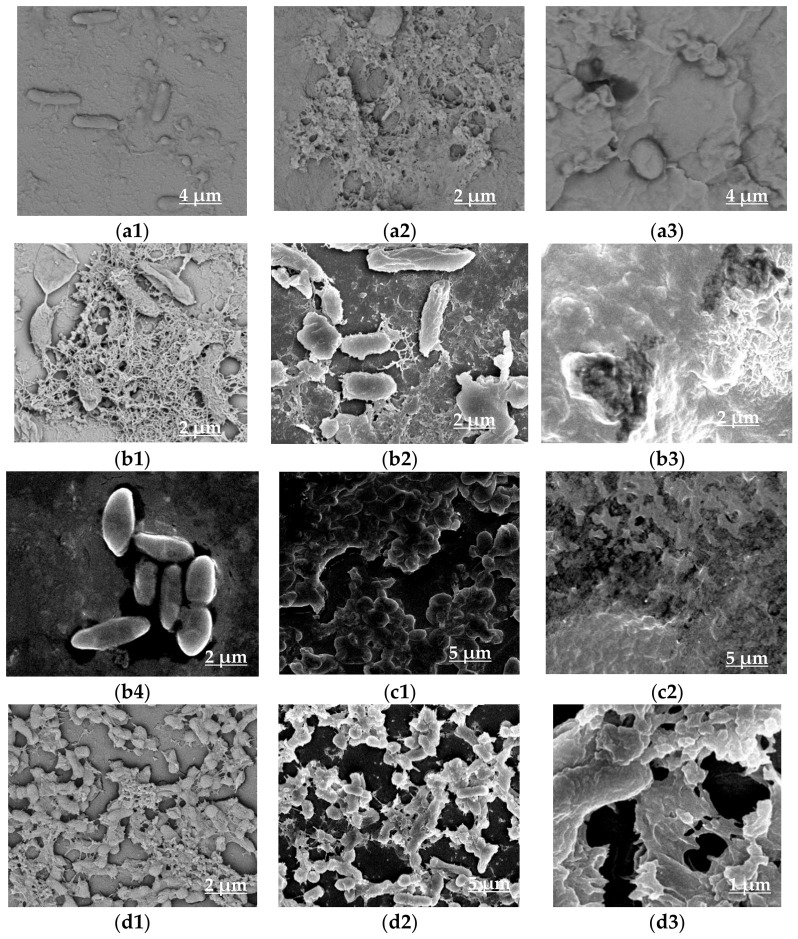
SEM image of the treated biofilms: (**a1**,**a2**) different zones and dimensions of a poly-species biofilm treated with a combination of a poly-hetero phage cocktail and ciprofloxacin and (**a3**) treated with ciprofloxacin alone. (**b1**,**b2**) different zones and dimensions of a dual-species biofilm of *P. aeruginosa* and *S. aureus* treated with a combination of a dual-hetero phage cocktail and ciprofloxacin, as well as (**b3**) treated with phage di-hetero-cocktail alone and (**b4**) treated with ciprofloxacin alone. (**c1**) a dual-species biofilm of *P. aeruginosa* and *K. pneumoniae* treated with a combination of a di-hetero phage cocktail and ciprofloxacin, and (**c2**) treated with a di-hetero phage cocktail alone. (**d1**–**d3**) the different zones and dimensions of a dual-species *S. aureus* and *K. pneumoniae* biofilm treated with a combination of a dual-hetero phage cocktail and ciprofloxacin.

The effects of the biofilm treatment can be observed using SEM images (Figure 11). We can see spatial disruption of the matrixes and reduced numbers of biomass.

**Figure 12:**
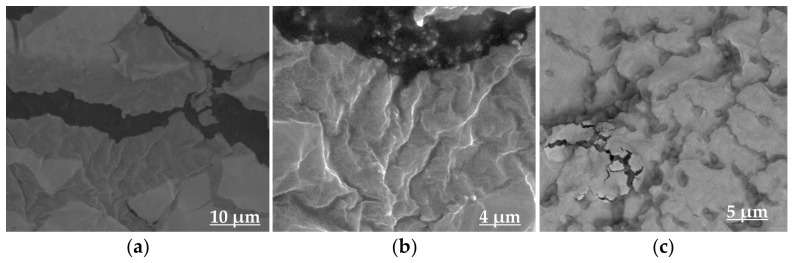
SEM images of the poly-species biofilms: (**a**–**c**): The different zones and dimensions treated with poly-hetero-cocktails.

Figure 12a–c shows SEM images of one-week-old poly-biofilms treated with poly-hetero-cocktails. We can particularly see the protective surface barrier being destroyed.

#### 2.3.8. Confocal Laser Scanning Microscopy (CLSM)

**Figure 13:**
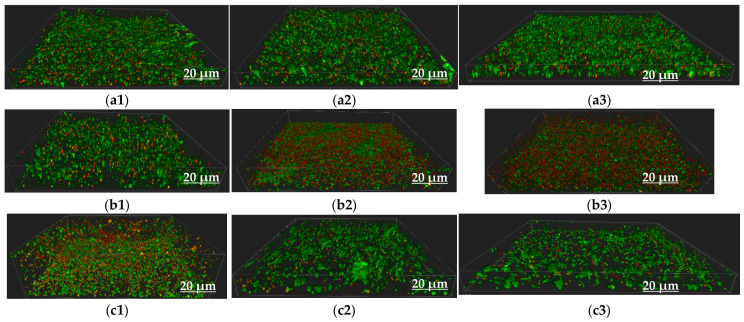
CLSM images of one-week poly-biofilms: (**a1**–**a3**) are different CLSM image captures treated with an antibiotic alone; (**b1**–**b3**) are different CLSM image captures treated with a phage poly-hetero-cocktail alone; and (**c1**–**c3**) are different CLSM image captures treated with a combination of the two. The biofilms were stained using the LIVE/DEAD™ BacLight™ Bacterial Viability Kit. Live cells stain green and dead cells stain red.

The architecture and bacterial biomass of the one-week poly-biofilms treated with a ciprofloxacin, a phage poly-hetero-cocktail, and with a combination of the two were visualized using confocal laser scanning microscopy (CLSM) (Figure 13): we can observe the effects of the (Figure 13(c1–c3) phage and (Figure 13(d1–d3) poly-hetero-cocktail both alone and in combination with ciprofloxacin on the poly-species biofilm. Ciprofloxacin alone (Figure 13(a1–a3) demonstrates a lesser reduction effect.

### 2.4. Testing the Sensitivity of Biofilm-Associated Bacterial Isolates to Ciprofloxacin

#### 2.4.1. Biofilm-Associated Antibiotic-Resistant Mutants Isolates

We attempted to isolate antibiotic-resistant mutants from different biofilm model sets and succeeded only with *P. aeruginosa* PAO1K sampled from a poly-species biofilm treated with combination of ciprofloxacin and phage poly-hetero-cocktail, which exhibited a change in sensitivity to resistance (from 0.25 µg/mL H to 1 µg/mL R, biofilm isolate (2)). The tests were performed using VITEK 2 (Ref. 27226, BioMérieux, Inc., USA), which was performed at the QAMH (Queen Astrid Military Hospital) Military Medical Laboratory Capacity (MMLC) ISO 15189 (Table 4). The official testing results from the MMLC laboratory are provided in the Appendix (Figure S5).

Furthermore, we isolated a *P. aeruginosa* PAO1K strain from a poly-species biofilm that was treated with a combination of ciprofloxacin and a poly-hetero-cocktail of phages. The MIC value decreased from 0.25 µg/mL to 0.12 µg/mL (Table 4, biofilm isolate (1)). Thus, the *P. aeruginosa* strain was resensitized in the context of a phage–antibiotic combination. The results of the MMLC laboratory testing are provided in Supplementary Figure S6.

#### 2.4.2. Biofilm-Associated Isolates: Morphological Characterization and Persistence

It is important to note that when samples from experimental biofilms were subjected to an enumeration assay, *S. aureus* and *P. aeruginosa*, which were sampled from an untreated dual-biofilm containing either *P. aeruginosa* and *S. aureus* or *S. aureus* and *K. pneumoniae*, and *P. aeruginosa* and *K. pneumoniae*, respectively, appeared to be countable after 48–72 h. Furthermore, *P. aeruginosa* isolates from ciprofloxacin-treated dual- and poly-species biofilms appeared countable after 48 h. *K. pneumoniae* biofilm isolates exhibited phenotypic heterogeneity, displaying both normal-sized and small colony variants (SCVs) (Figure S7), which is also a survival strategy. The late appearance of *S. aureus* and *P. aeruginosa* could also be attributed to SCVs, which are optimized for persistence [52].

## 3. Discussion

Biofilm-associated infections account for 65% to 80% of all microbial infections in humans and are a global crisis [19]. These dynamic, complex bacterial communities are surrounded by an extracellular polymeric matrix that shields pathogens from antimicrobials. The emergence of multidrug-resistant (MDR) pathogens necessitates an urgent solution: an anti-biofilm treatment strategy. Phage–antibiotic synergistic therapy is considered one of the most promising anti-biofilm strategies. Phages can target different stages of biofilm formation.

**Figure 14:**
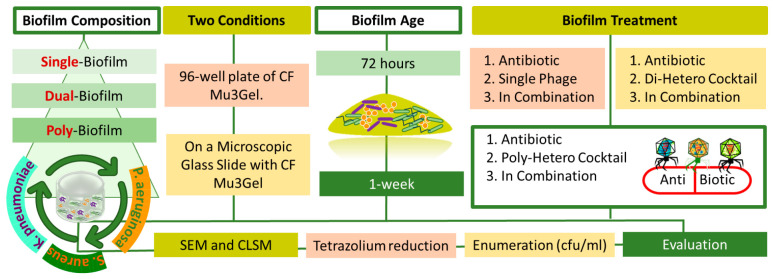
Scheme of Biofilm Development and Treatment.

In nature, biofilms typically comprise multiple species [7]. Interspecies communication defines their spatial structure, as well as the composition and relative abundance of their subpopulations within the total microbial biofilm. This process is crucial for advancing biofilm-associated infections. Mixed infections of *P. aeruginosa* with *S. aureus* and *K. pneumoniae* are often present in severe cases of community-acquired pneumonia. The strains used here—PAO1K, ATCC 6538, and ATCC 27736, respectively—are known for their strong, stable, and structured ability to form biofilms [17,53,54,55]. However, it is very challenging to mimic natural conditions and maintain a long-term, balanced community of these pathogens in a laboratory setting [5]. The CF-Mu3Gel tested in this study is a dynamic and heterogeneous structure consisting of smooth and wrinkled shells, hills with a mushroom-like appearance [50], valleys, and channels that mimic the human body’s physiological environment. Figure 14 shows the study structure and the flow of the experiments. This enabled the results of the various criteria and values to be compared, to provide an overall evaluation.

Poly-species biofilms exhibit cooperative or competitive interactions and establish a communication network controlled by specific genes and signals, resulting in compositional equilibrium [7]. In the biofilm models studied here, the biomass balance of individual species in dual- and poly-biofilms remained stable throughout the three-day to one-week incubation period due to a decrease in one species and an increase in another, in addition to competitive interactions (Figure 6). Additionally, the relative abundance of dominant components in different dual-species models is decreased by about one log compared to single biofilms. Therefore, the interactions between the components were considered cooperative. Pan et al. describes that *P. aeruginosa* often exhibits cooperative behavior in biofilms by secreting EPS and siderophores that benefit co-cultured pathogens [5,56,57]. Additionally, staphylococcal protein A (SpA) interacts with *P. aeruginosa*, promoting aggregation via Psl and type-IV pilus binding [58,59]. In a dual-species biofilm of *S. aureus* and *P. aeruginosa*, the last dominates *S. aures* by only about one log.

Woods et al. developed a dual-species biofilm of *S. aureus* and *P. aeruginosa* in a silicone tube reactor system at 22 °C under continuous-flow conditions. Interestingly, they used the same strains (PAO1 and ATCC 6538) as in this study, but different versions, as they had been maintained in different laboratories for years. They demonstrated that *S. aureus* persisted at higher concentrations within dual-species biofilms containing *P. aeruginosa* [4]. We also observed *S. aureus* persistence in dual-species biofilms containing either *P. aeruginosa* or *K. pneumoniae*. It is also important to note that *S. aureus* biomass counts increased by one log over one week in the poly-species biofilm, while *P. aeruginosa* and *K. pneumoniae* loads decreased by one log. Furthermore, during the CFU/mL enumeration assay, *S. aureus* colonies were observed to appear after 48–72 h in untreated dual-species biofilms (Figure S7). This finding could explain the persistence of *S. aureus*. Studies performed with metabolic regulators have shown that reduced metabolic activity is correlated with increased persistence [52]. Although a reduced metabolic state is associated with dormancy, it can also contribute to persistence. Persister cells are not necessarily dormant and may employ alternative survival mechanisms [33,60]. The “triggered persisters” could be formed in response to environmental stressors, including reactive oxygen species and quorum-sensing molecules (QS) [33]. Persistence may represent a continuous quantitative trait rather than a binary ‘persister–non-persister’ state. This would be consistent with it being a complex polygenic feature [33]. However, we observed the same phenomenon with *P. aeruginosa* in both untreated and ciprofloxacin-treated biofilms. In the latter case, the strain ratios were altered, with *S. aureus* dominating *P. aeruginosa* (Figure 8). This led us to conclude that *S. aureus* microcolonies consisted of a mixture of persistent and antibiotic-tolerant cells. ‘Triggered persisters’ are characterized by the highest level of antibiotic tolerance within a population exhibiting varying levels of susceptibility due to developmental noise in various tolerance mechanisms [33]. Furthermore, we presume that the persistent *S. aureus* and *P. aeruginosa* cells required time to recover and become visible on an agar plate [61,62]. Persister cells need to initiate the recovery and regrowth process, which can be triggered by various environmental factors, such as fresh nutrients and quorum-sensing (QS) signals from growing cells [33].

Furthermore, *K. pneumoniae* biofilm isolates exhibited phenotypic heterogeneity, displaying both normal-sized and small colony variants (SCVs). This is also a survival strategy related to increased biofilm production and antibiotic tolerance. Additionally, *S. aureus* and *P. aeruginosa* demonstrate increased antibiotic persistence due to the secretion of signaling molecules [33]. We encountered this with the *P. aeruginosa* PAO1K biofilm isolate, which exhibited a change in sensitivity to resistance (from 0.25 µg/mL H to 1 µg/mL R) (Table 5). Although we did not succeed in isolating a ciprofloxacin-resistant strain of *S. aureus* due to limited testing, *S. aureus*’s increased antibiotic persistence is demonstrated by its domination over *P. aeruginosa* and *K. pneumoniae* under exposure to ciprofloxacin, either alone or in combination. Another phenomenon was observed with *S. aureus* and *K. pneumoniae* single-, dual-, and poly-species planktonic cultures. These cultures produced the same RRU reduction curves at concentrations ranging from 1 to 8 µg/mL over a period of up to 24 h (Figure S3). This phenomenon could be described as a plateau in inhibition [63], where higher concentrations of the drug no longer increase killing power. The same phenomenon could occur in biofilms with the same compositions if we translate these planktonic cultures’ results into biofilms. Once again, this often indicates that a subpopulation of bacteria, such as persister cells, has survived, or that the bacteria have developed tolerance to the treatment. The terms “antibiotic persistence” and “antibiotic tolerance” are often used interchangeably. Both terms describe an increased survival rate in the presence of an antibiotic without a change in the MIC [64]. Salcedo-Sora et al. also reported that two of the fluoroquinolones (ciprofloxacin and ofloxacin) and gentamicin reveal the “Eagle effect”, where higher concentrations of antibiotic can have a reduced effect [63].

*K. pneumoniae* biomass was more abundant than any other component in untreated, 72-h, dual-species biofilms containing *P. aeruginosa* and *S. aureus*. However, the same degree of dominance of *K. pneumoniae* was observed in the one-week dual-biofilm with *S. aureus*, while decreasing by 1.5 logs in the dual-biofilm with *P. aeruginosa*. Muzaki et al. described dual-species biofilms of *K. pneumoniae* and *P. aeruginosa* forming two distinct layers, with *K. pneumoniae* occupying the top layer and growing over a layer of *P. aeruginosa* biofilm. However, the distinct, dual-layer arrangement of biofilms could be altered by spatial arrangement. That could happen due to changes made during biofilm development, which correspondingly change the proportion of pathogens. In our case, the proportion of *P. aeruginosa* to *K. pneumoniae* increased due to a decrease in *K. pneumoniae* load in one log, and the *P. aeruginosa* biomass load remained stable for up to one week. The decrease in *K. pneumoniae* compared to *P. aeruginosa* could be Autoinducer-2 (AI-2) mediated due to the lowered volume of basal *P. aeruginosa* needed for *K. pneumoniae* to attach to and mature on [7].

The ability of CF-Mu3Gel to facilitate cell drifting promoted the development of spatial structures in the poly-species biofilms. This benefited the growth of *S. aureus* biomass while preventing the overgrowth of *P. aeruginosa* and *K. pneumoniae*, as evidenced by the decrease in their biomass. Additionally, it was reported that *K. pneumoniae* acts as a “bridging organism” within polymicrobial biofilms, mediating interspecies interactions through Autoinducer-2. (AI-2) [7]. The domination of *P. aeruginosa* to *K. pneumoniae* could be explained differently; for example, in iron-limited conditions, *P. aeruginosa* outfits *K. pneumoniae* by employing rhamnolipid biosurfactant [5,65]. However, it could also be caused by the AI-2-mediated decrease in biofilm formation in *K. pneumoniae*, as described above [7].

Forti et al. reported that treatment with *P. aeruginosa* phages, both alone and in a phage cocktail, can enter biofilms and destroy biomass, including different communities formed by different CF infection isolates [20]. We propose that the high 3-log reduction observed with combination therapy in the 72-h single-species biofilms of *P. aeruginosa* is due more to ciprofloxacin, as ciprofloxacin alone results in a 2.5-log reduction. The biofilm models in which the phage or phage cocktail exhibited higher activity than ciprofloxacin include 72-h dual-species biofilms of *P. aeruginosa* and *S. aureus*, as well as poly-species biofilms from both the 72-h and one-week models.

Subinhibitory concentrations of ciprofloxacin stimulate *S. aureus* biofilm formation via an agrC-dependent pathway, which could facilitate bacterial persistence [66]. Ciprofloxacin resulted in increased biofilm formation and higher bacterial loads in *S. aureus* and *K. pneumoniae* dual biofilms compared to the control. This is primarily due to phenotypic tolerance, whereby the entire population, not just mutants, enters a state of reduced growth [63]. The change in the proportion of bacterial strains after treatment was primarily due to exposure to ciprofloxacin, with *S. aureus* dominating *P. aeruginosa* and *K. pneumoniae* to a lesser extent (Figure 8). It has also been reported that the quorum-sensing system controls the ratio of fast-growing to slow-growing, antibiotic-tolerant states [33].

Canfield et al. demonstrated that the evolution of phage resistance results in fitness defects and drug sensitization [67,68]. Furthermore, we observed this intriguing phenomenon in a *P. aeruginosa* PAO1K poly-species biofilm isolate treated with a combination of ciprofloxacin and a poly-hetero-cocktail of phages. Its MIC value decreased from 0.25 µg/mL to 0.12 µg/mL (Table 5). Thus, the *P. aeruginosa* strain was resensitized by the phage–antibiotic combination.

However, the CF Mu3Gel three-dimensional biofilm structure consists of microbial communities and microcolonies of different ages and species in varying proportions (Figure 4), embedded in an EPS matrix. This could be referred to as “spatial vulnerability” [69], Thus, areas with more metabolically active bacteria could support phage amplification [51]. CLSM images of biofilms treated with the phage poly-hetero-cocktail alone (Figure 13(b1–b3)) or in combination with ciprofloxacin (Figure 13(c1–c3)) reveal the spatial nature of the treatment. This spatial effect can also be observed in SEM images of the combination treatment (Figure 11). Additionally, the mechanistic phenomena causing individual cells to grow slowly in an otherwise fast-growing population would necessarily involve systems with at least two partners that switch the cells between growth and non-growth states [70].

Furthermore, we presume that the phages were initially actively propagating in the surface layer of the biofilms, because they prefer less mature microcolonies [51]. We confirmed this by revealing an increase in *P. aeruginosa* and *S. aureus* phage titers compared to the initial inoculum, whether the phages were used alone or in a cocktail (Figure 10). We hypothesize that some phage particles are mechanically spread to relatively deeper biofilm layers. This could be facilitated by the hollow, spatial structures of the mature biofilm and by the capacity of CF Mu3Gel, which facilitates pathogens to drift and colonize the full thickness of the gel [45]. Additionally, examining several SEM images (Figure 12a–c) of one-week poly-biofilms treated with poly-hetero-cocktails reveals areas of the biofilm with a compromised surface barrier. We hypothesize that this could enable phages and antibiotics to penetrate the biofilm. Phage particles released from biofilm surfaces could spread to bacteria located further away [23]. Breaches in the biofilm could also facilitate the formation of channels that reestablish an environment conducive to the growth of fast-growing cells. This could enhance the effectiveness of phages or phage cocktails.

CLSM images of the treated biofilm also clearly demonstrate the lytic activity of the phage poly-hetero-cocktail in a one-week poly-biofilm (Figure 13(c1–c3)), as confirmed by the CFU/mL evaluation test. Web et al. observed that cell death was associated with the temporal and spatial organization of the biofilm [71]. Inside microcolonies, up to 50% of *P. aeruginosa* biofilms showed killing and lysis in their centers after two weeks of experiments. These results are consistent with our CLSM images, which show a network of live and dead bacterial cells spread throughout the untreated biofilms, likely in balance (Figure 5).

## 4. Materials and Methods

### 4.1. Bacterial and Bacteriophage Strains

This study used the following bacterial strains: *Pseudomonas aeruginosa* PAO1K (ATCC 15692); *Staphylococcus aureus* ATCC 6538 (FDA 209); and *Klebsiella pneumoniae* ATCC 27736. All three strains were preserved in the bacterial strain collection at the Laboratory for Molecular and Cellular Technology (LabMCT) at the Queen Astrid Military Hospital (QAMH) in Brussels, Belgium. The bacteriophage strains used were Atpa010 of and Atkp010 of *K. pneumoniae* from the LabMCT collection (GenBank accession no. PZ233457 and PZ233474, respectively), which have been previously studied and screened om 102 *P. aeruginosa* and 155 *K. pneumoniae strains* [46]; as well as Sb1 of *S. aureus* (GenBank accession no. HQ163896) [47], kindly provided by the G. Eliava Institute of Bacteriophages, Microbiology and Virology.

#### Bacterial Growth Media and Conditions

Lysogeny Broth (LB) (Lennox, L3022-1KG; Sigma-Aldrich, Burlington, MA, USA) was used for the bacterial and phage culture of *P. aeruginosa* and Tryptic Soy Broth (TSB) No. 2 (Millipore, Molsheim, France, 51228-500G-F) were used for *S. aureus* and *K. pneumoniae*, with or without the addition of bacteriological agar (VWR Chemicals, Suwanee, GA, USA, #9002-18-0).

Bacterial stocks were maintained in TSB broth containing 15% glycerol at −80 °C. Bacterial cultures were routinely prepared in LB broth and incubated overnight at 37 °C. Working bacterial suspensions were prepared in Dulbecco’s Phosphate-Buffered Saline (DPBS) (Lonza™ BE17-512F, Walkersville, MD, USA) and diluted to an optical density (OD600 using a PerkinElmer Lambda 12 UV/VIS spectrometer (PerkinElmer, Macquarie Park, NSW 2113, Australia)) corresponding to the desired colony-forming units per ml (cfu/mL). Adjustment of the OD to cfu/mL was performed, as previously described [46].

### 4.2. Antibiotic

Ciprofloxacin (Sigma-Aldrich, #17850) was used to treat the biofilm. A stock solution was prepared by dissolving the drug in sterile lysogeny broth (LB) to achieve a final concentration of 10 mg/mL.

#### Determination of the Minimal Inhibitory Concentration of Antibiotics

The minimal inhibitory concentration (MIC) was evaluated using the broth microdilution method, strictly in accordance with the European Committee on Antimicrobial Susceptibility Testing (EUCAST) guidelines [72]. Pure cultures of *P. aeruginosa*, *S. aureus*, and *K. pneumoniae* strains were standardized to 5.0 × 10^5^ colony-forming units (CFU)/mL. These cultures were then exposed to various concentrations of antibiotics (64 mg/L − 0.0625 mg/L) at 37 °C for 24 h in the OmniLog system, in triplicate. The OmniLog system monitored and recorded a reduction in the tetrazolium dye due to bacterial respiration, causing a color change, every 15 min. Bacterial growth was expressed in relative respiration units (RRUs). After the incubation period, bacterial growth for each antibiotic concentration was assessed by analyzing bacterial growth curves with Biolog Data Analysis 1.7 software. The MIC value was identified as the lowest concentration of the antimicrobial molecule required to inhibit visible growth of the tested strain.

The MICs that we calculated were validated using the VITEK 2 (Ref. 27226, BioMérieux, Inc., USA) at the QAMH Military Medical Laboratory Capacity (MMLC) (ISO 15189).

### 4.3. Phage Lytic Activity Evaluation Using Phage Liquid Culturing (PLC)

The evaluation of phage lytic activity in all relevant experiments was performed as previously described [46]. According to the Appelmans protocol, the optimal phage-to-bacteria ratio was predetermined. A particular bacterial strain at a specific dilution was used in a 96-well microtiter plate. The plates were prepared as follows: First, 180 μL of the bacterial suspension in LB medium was added to each well except the “phage only” and “LB only” wells. Then, 20 μL of a phage (or phage hetero-cocktail) suspension at a given dilution was added to each test and “phage only” control well. Lastly, 100% (*v*/*v*) Redox Dye Mix H (100X) (Biolog #74228) was added to all wells, yielding a final concentration of 1%. The plate layout included “phage only,” “bacteria only,” and “LB only” controls. All experiments and controls were performed in triplicate wells. The test plates were then immediately incubated in the OmniLog system (Biolog, Hayward, CA, USA) at 37 °C for 48 h. A possible reduction was analyzed using Biolog Data Analysis 1.7 software to produce bacterial growth curves.

### 4.4. Biofilm Formation

A Cystic Fibrosis Model Mucus Gradient Air3Gel (Bac3Gel, Taguspark, Av. Jacques Delors 411 Lab 2; 2740-122 Porto Salvo, Portugal; https://www.bac3gel.com (accessed on 15 May 2026)) in a 96-well plate (#20250103A3GCF) and a 5-mL syringe (#20250604A3GCFG) were used separately for biofilm formation. Single-, dual-, and poly-biofilms of *P. aeruginosa*, *S. aureus*, and *K. pneumoniae* were developed over two time periods: 72 h and one week. The biofilms were developed under two conditions: in a 96-well plate with 100 µL of CF Mu3Gel in each well, and on microscope glass slides measuring 26 × 76 mm (#1169222, Knittel Glasbearbeitungs GmbH; Varrentrappstr. 5; 38114 Braunschweig, Germany) with 200 µL of CF Mu3Gel.

In the latter case, 200 µL of CF Mu3Gel was dispensed from a syringe onto the center of a sterile microscope slide. The slide was then placed in a round Petri dish (#688161, Greiner, Noida, India). For each condition, an overnight culture of each bacterial strain was diluted in LB broth to a concentration of 10^5^ CFU/mL. Then, 100 µL and 200 µL of the suspension was inoculated into each well of a 96-well plate containing CF Mu3Gel, as well as onto 200 µL of CF Mu3Gel on a microscope glass slide. The slides were then incubated for the desired time period at 37 °C. For dual- and poly-biofilms, the *S. aureus* culture was inoculated for 24 h before inoculating the *P. aeruginosa* and/or *K. pneumoniae* cultures. This allowed *S. aureus* to establish itself in the pre-formed biofilm and ensured that it would coexist with *P. aeruginosa* and *K. pneumoniae* rather than be rapidly eradicated by them. For each day of the experiment, 100 and 200 µL of LB broth was added to each well and to the surface of the microscopic glass biofilm for 72 h and one week, respectively.

### 4.5. Treatment of Established Biofilm

Before treatment, planktonic cells were removed from 72-h and one-week biofilms in a 96-well plate or on microscope slides. The biofilms were then washed twice with DPBS. The biofilms were then exposed to one of the following treatments: broth (for biofilm growth control), an antibiotic, a phage, a phage di-hetero-cocktail (at a 1:1 ratio), a phage poly-hetero-cocktail (at a 1:1:1 ratio), or a combination of an antibiotic and a phage hetero-cocktail (at a 1:1 ratio), A total of 100 µL and 200 µL of the treatment suspension was added to the CF Mu3Gel, respectively (Table 5). The plates were incubated for 24 h at 37 °C.

### 4.6. Biofilm Reduction Evaluation

#### 4.6.1. Enumeration of Bacteria and Phage upon Completion of Biofilm Treatment

The planktonic cells were removed from the 96-well plate or the microscopic glass slides. The biofilms were treated with a Bac3Gel Dissolution Medium (B3G-DM) for 5 min to facilitate the separation of bacterial cells.

Then, the biofilms were dislodged mechanically and homogenized by vigorous pipetting. Bacterial enumeration (cfu/mL) was performed as previously described [46]. Initially, 20 μL was aspirated from each test well (in triplicate) using a multichannel pipette. Then, the contents were transferred to a 96-well microtiter plate containing 180 μL of DPBS. The well contents were serially diluted 10-fold, ranging from 0 to 7. These dilutions (20 μL) in triplicate were then spotted in five drops across a column of the plate (square Petri dish measuring 120/120/17 mm) using a multichannel pipette on the solid agar surface. The plates were incubated upside down at 37 °C for 18 h. The average number of individual colonies for each dilution was calculated to determine the CFU/mL. The same dilutions were used for phage particle enumeration using the MD/SP (Multiple Dilutions on Single Plates) method [73].

#### 4.6.2. A Tetrazolium Reduction Curve in the OmniLog System

The OmniLog system was only used to analyze 72-h biofilms that had been developed in 96-well plates. The first biofilm was allowed to develop without dye for the required incubation period. The mature biofilms were then treated with antimicrobials in the absence of dye. After six hours, the redox dye was introduced for an additional 18 h to observe the reduction in bacterial cells compared to the control. The results were interpreted as described in the Section Determination of the Minimal Inhibitory Concentration of Antibiotics.

#### 4.6.3. Enumeration of Bacteria in a Selective Environment Using Phages as Inhibitory Agents

A volume of 300 µL of a phage suspension at a concentration of 2.0 × 10^10^ pfu/mL was placed in the center of a square Petri plate and spread evenly across the surface using a disposable spreader. After drying, further dilutions and counts (CFU/mL) were performed as described in Section 4.6.1.

### 4.7. Scanning Electron Microscopy (SEM)

For this study, only biofilms on microscopic slides were used. The samples were rinsed twice in 1 mL of PBS (Phosphate-Buffered Saline) for one minute each and left to dry. Then, 1 mL of a 2.5% glutaraldehyde (#3778.1, ROTH) solution in a 0.1 M cacodylate buffer (#5169.3, ROTH) solution (pH 7.4) was added to the biofilm. The biofilm was then incubated for 30 min. The glutaraldehyde solution was removed, and the biofilm was rinsed with 1 mL of PBS three to four times for five minutes each. The biofilm was dehydrated with serial washes of 1 mL each of 30%, 50%, 70%, and 90% ethanol for 20 min. Then, the biofilm was soaked in 1 mL of 100% ethanol three times for 20 min each.

The fixed biofilms on the discs were sputter-coated with a 5-nm platinum coating (Q150T ES Plus, Quorum Technologies, East Sussex, UK) and observed using a scanning electron microscope (SEM) operated in high-vacuum settings with a high-resolution in-lens secondary electron (SE) detector (magnetic immersion lens) or backscattered electron images. A low accelerating voltage of 5 keV was applied to minimize damage to the biofilm matrix from the beam. The working distance and spot size were set to 5 mm and 3 mm, respectively.

### 4.8. Confocal Laser Scanning Microscopy (CLSM) Visualization

We evaluated biofilm viability following the different treatments using CLSM. Biofilms developed on glass microscope slides were stained with the LIVE/DEAD™ BacLight™ Bacterial Viability Kit (L34856, Invitrogen, Thermo Fisher Scientific, 5791 Van Allen WayCarlsbad, CA 92008, USA) for 20 min at room temperature in the dark, according to the manufacturer’s instructions. Then, the staining solution was carefully removed and the biofilm samples were transferred to a 24-well glass-bottom plate with a high-performance #1.5 cover glass (#P24-1.5H-N, Cellvis LLC, Mountain View, CA 94039, USA). Fully hydrated biofilms were imaged using a Leica TCS SP8 inverted confocal laser scanning microscope (Leica Microsystems, GmbH, Ernst-Leitz-Strasse 17-37; 35578 Wetzlar, Germany), which was equipped with a 63× glycerol immersion objective. For each experimental condition, 8-bit z-stacks (1024 × 1024 pixels) were acquired from at least three randomly selected locations per biofilm. Optical sections were collected with a z-step size of 1 µm. The image stacks were visualized and processed using ImageJ 13.0.6 (National Institutes of Health, Bethesda, MD, USA), and three-dimensional biofilm reconstructions were generated using Imagis Viewer (Bitplane, Oxford Instruments, Zürcherstrasse 6, 8952 Schlieren, Switzerland).

### 4.9. Statistical Analyses

Statistical analyses were performed using Prism 11.0.0. (GraphPad Software, Boston, MA, USA). The results are shown as mean with SD, and *p* < 0.05 was considered statistically significant.

## 5. Conclusions

The single-, dual-, and poly-species 3D biofilms of *P. aeruginosa*, *S. aureus*, and *K. pneumoniae* that we developed in CF Mu3Gel were complex and dynamic, consisting of a high relative abundance of microcolonies of different species. Demonstrating synergistic interactions, the results show each component’s ability to persist in dual- and poly-species biofilms, regardless of treatment, and how phages can modify biofilm composition. This could counteract the persistent and resilient nature of biofilm-associated pathogens and cause re-sensitization to antibiotics. Thus, these biofilms mimic the physiological settings of the human body.

This study highlights the potential of combining a phage, a phage di-hetero-cocktail, or a phage poly-hetero-cocktail with an antibiotic to enhance the treatment of single-, dual-, and poly-biofilms of *P. aeruginosa*, *S. aureus*, and *K. pneumoniae* pathogens. The results clearly demonstrate synergy between the phages in the hetero-cocktail and the antibiotic.

In the future, these 3D biofilm models could be used to screen phages or phage cocktails, either alone or in combination with other therapies. This would facilitate translating in vitro experience into real physiological settings, developing strategies for multiple follow-up treatments, and reordering phages, phage cocktails, and antibiotic combinations, with the goal of improving outcomes.

## Figures and Tables

**Table 1:** Load of each bacterial strain in 72-h and one-week biofilm models (CFU/mL).

Bacterial Strains	Dual-Species Biofilms (cfu/mL)				Poly-Species Biofilm (cfu/mL)			
	*P. aeruginosa* *S. aureus*		*P. aeruginosa* *K. pneumoniae*		*S. aureus* *K. pneumoniae*		*P. aeruginosa, S. aureus,* *K. pneumoniae*	
	72 h	One-Week	72 h	One-Week	72 h	One-Week	72 h	One-Week
*P. aeruginosa*	5.85 × 10^8^	3.45 × 10^8^	1.21 × 10^6^	3.53 × 10^6^	n/a	n/a	2.40 × 10^9^	8.10 × 10^8^
*S. aureus*	2.23 × 10^7^	6.10 × 10^6^	n/a	n/a	5.07 × 10^5^	1.12 × 10^5^	2.33 × 10^4^	1.20 × 10^6^
*K. pneumoniae*	n/a	n/a	1.66 × 10^9^	2.37 × 10^8^	4.13 × 10^8^	2.27 × 10^8^	1.23 × 10^8^	4.60 × 10^7^

n/a—not applicable.

**Table 2:** Reduction values for bacterial load after treatment of 72-h and one-week old biofilms for 24 h. The values are means ± SD of triplicates (*n* = 3).

Single-, Dual-, and Poly-Species Biofilm	72 h (CFU/mL)			One Week (CFU/mL)		
	Ciprofloxacin	Phage, Phage Cocktail	Combination	Ciprofloxacin	Phage, Phage Cocktail	Combination
*P. aeruginosa*	4.17 × 10^2^	5.70 × 10^1^	1.02 × 10^3^	2.82 × 10^1^	7.82 × 10^0^	1.09 × 10^1^
*S. aureus*	1.11 × 10^1^	1.92 × 10^1^	6.94 × 10^1^	6.71 × 10^−1^	4.56 × 10^0^	7.34 × 10^0^
*K. pneumoniae*	2.31 × 10^1^	6.79 × 10^0^	3.76 × 10^1^	1.26 × 10^0^	1.59 × 10^0^	2.35 × 10^0^
*P. aeruginosa* and *S. aureus*	1.37 × 10^1^	1.67 × 10^2^	1.57 × 10^2^	3.87 × 10^0^	6.13 × 10^0^	7.85 × 10^0^
*P. aeruginosa* and *K. pneumoniae*	5.25 × 10^0^	2.02 × 10^0^	7.64 × 10^0^	3.40 × 10^0^	3.3 × 10^0^	3.63 × 10^0^
*S. aureus* and *K. pneumoniae*	4.09 × 10^0^	4.87 × 10^0^	8.68 × 10^0^	6.04 × 10^−1^	2.04 × 10^0^	2.03 × 10^0^
*P. aeruginosa*, *S. aureus* and *K. pneumoniae*	9.63 × 10^0^	5.20 × 10^1^	6.05 × 10^1^	1.52 × 10^0^	1.15 × 10^1^	4.09 × 10^0^

■ The high synergy effect is shown in dark green; ■ the medium synergy effect is shown in medium green; ■the low synergy effect is shown in light green; ■ the proto-cooperation effect is shown in light golden; ■ and the negative effect of antibiotic alone is shown in light orange.

**Table 3:** Overall interpretation of the results of the single-, dual-, and poly-species biofilm treatments.

Components of Biofilms	Interpretation of the Results
*P. aeruginosa*	In the single biofilm of *P. aeruginosa*, the combination therapy resulted in a 3-log reduction with a high synergistic effect in a 72-h biofilm and a 1-log reduction with a proto-cooperative effect in a one-week biofilm.
*S. aureus*	In the single biofilm of *S. aureus*, there was a 1.8-log reduction with a high synergistic effect in a 72-h biofilm and a 0.87-log reduction with a medium synergistic effect in a one-week biofilm. The ciprofloxacin alone had a negative effect, resulting in an increase in bacterial load.
*K. pneumoniae*	In the single biofilm of *K. pneumoniae*, there was a 1.6-log reduction and a high synergistic effect after 72 h, but after one week, there was a minor 0.4-log reduction with a negligible synergistic effect.
*P. aeruginosa* and *S. aureus*	In the dual-species biofilm of *P. aeruginosa* and *S. aureus*, there was a 2.2-log reduction with proto-cooperation effect in the 72-h biofilm and a 0.9-log reduction with a low synergistic effect in the one-week biofilm.
*P. aeruginosa* and *K. pneumoniae*	In the dual-species biofilm of *P. aeruginosa* and *K. pneumoniae*, there was a 0.9-log reduction with a medium synergistic effect in the 72-h biofilm and a 0.56-log reduction in the one-week biofilm with the proto-cooperation effect.
*S. aureus* and *K. pneumoniae*	In the dual-species biofilm of *S. aureus* and *K. pneumoniae*, there was a 0.94 log reduction with a medium synergistic effect in the 72-h biofilm and a low 0.3-log reduction in the one-week biofilm. This reduction was due to the phage; the ciprofloxacin alone had a negative effect, resulting in an increase in bacterial load.
*P. aeruginosa*, *S. Aureus*, and *K. pneumoniae*	In a poly-species biofilm, a 1.78-log reduction with a negligible synergy effect in the 72-h biofilm and a 0.6-log reduction in the one-week biofilm, which is lower than a tri-hetero-cocktail effect in a single.

**Table 4:** Interpretation of the results of the single-, dual-, and poly-species biofilm treatment.

Bacterial Strains	Original Strains (µg/mL)	Biofilm Isolates (1) (µg/mL)	Biofilm Isolates (2) (µg/mL)
*P. aeruginosa* PAO1K	0.5 H ^1^	0.5 H	0.5 H
*S. aureus* 6538	0.25 H	1.0 R	0.12 H
*K. pneumoniae* ATCC 27736	0.06 S ^2^	0.06 S	0.06 S

^1^ H—intermediate; ^2^ S—Sensitive.

**Table 5:** The formulations of phage, ciprofloxacin, and their combination, in 100 µL and 200 µL suspensions, were applied to treat biofilm in individual wells of a 96-well plate and on microscopic glass slides.

Inoculum Composition	CF Mu3Gel Biofilm Establishment Condition	
	100 µL in a 96-Well Plate	200 µL on a Microscope Glass Slide
Phage (PFU/mL)	100 µL of a 2.0 × 10^10^	200 µL of phage of 2.0 × 10^10^
Phage di-hetero-cocktail (PFU/mL)	50 µL of each phage of 4.0 × 10^10^	100 µL of each phage of 4.0 × 10^10^
Phage tri-hetero-cocktail (PFU/mL)	33 µL of each phage of 6.0 × 10^10^	66.6 µL of each phage of 6.0 × 10^10^
Ciprofloxacin (µg/mL)	100 µL of Ciprofloxacin of 8	200 µL of Ciprofloxacin of 8 µg/mL
Phage (PFU/mL) ciprofloxacin (µg/mL)	50 µL of phage 4.0 × 10^10^ 50 µL of ciprofloxacin 16	100 µL of phage of 4.0 × 10^10^ 100 µL of ciprofloxacin 16
Phage di-cocktail (PFU/mL) Ciprofloxacin (µg/mL)	25 µL of each phage of 8.0 × 10^10^ 50 µL of ciprofloxacin 16	50 µL of each phage of 8.0 × 10^10^ 100 µL of ciprofloxacin 16
	16.6 µL of each phage of 1.2 × 10^11^ 50 µL of ciprofloxacin 16	33.3 µL of each phage of 1.2 × 10^11^ 100 µL of ciprofloxacin 16

## Data Availability

The original contributions presented in this study are included in the article/Supplementary Materials. Further inquiries can be directed to the corresponding author.

## Supplementary Materials

The following supporting information can be downloaded at: https://www.mdpi.com/article/10.3390/antibiotics15060537/s1, Figure S1: Lytic activity of phages and phage cocktails in low-load planktonic cultures; Figure S2: Antibiotic sensitivity test validated by the VITEK 2 and performed at the QAMH Military Medical Laboratory Capacity (MMLC). Figure S3: The proliferation curves of the high concentration planktonic cultures. Table S1: Reduction of High-Load Planktonic Cultures and Co-Cultures upon Exposure to 1–8 µg/L of Ciprofloxacin. Figure S4: Lytic activity of phages and phage cocktails in high-load planktonic cultures. Figure S5: The antibiotic sensitivity re-test (1), performed using the VITEK 2 at the QAMH Military Medical Laboratory Capacity (MMLC. Figure S6: The antibiotic sensitivity re-test (2), performed using the VITEK 2 at the QAMH Military Medical Laboratory Capacity (MMLC). Figure S7: Pictures of enumeration plates.
