# Supplementary material for: Single Treatment of Mature 3D Single-, Dual- and Poly-Species Biofilms Using a Combination Therapy of Phage or Phage-Hetero-Cocktails and Ciprofloxacin
Source: Antibiotics (Basel). 2026 May 25;15(6):537. doi: 10.3390/antibiotics15060537 (PMC13295384; doi:10.3390/antibiotics15060537)
Supplement: Supplementary file 1 [file antibiotics-15-00537-s001.zip › antibiotics-4282580-supplementary.pdf]

Supplementary

Figure S1. Lytic activity of phages and phage cocktails in low-load planktonic cultures: (a) *P. aeruginosa* phage Atpa010, *S. aureus* phage Sb1, and *K. pneumoniae* phage Atkp010. (b) Phage di-cocktails: Atpa010 and Sb1; Atpa010 and Atkp010; Sb1 and Atkp010; and tri-cocktail: Atpa010, Sb1, and Atkp010. Values are the mean ± SD of triplicates (n = 3).

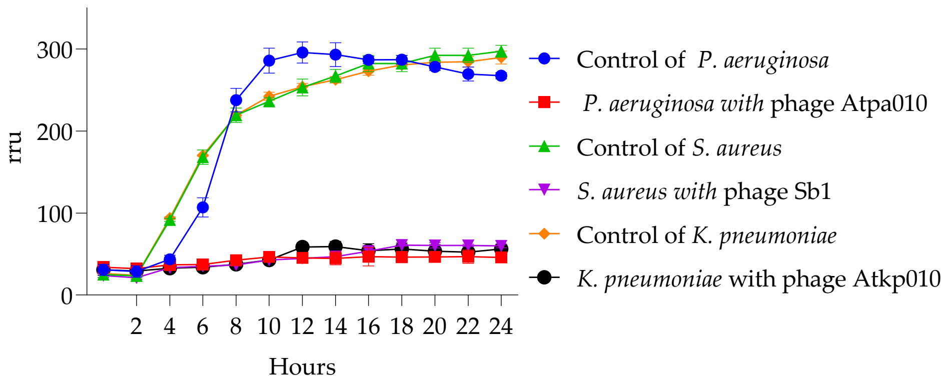

(a)

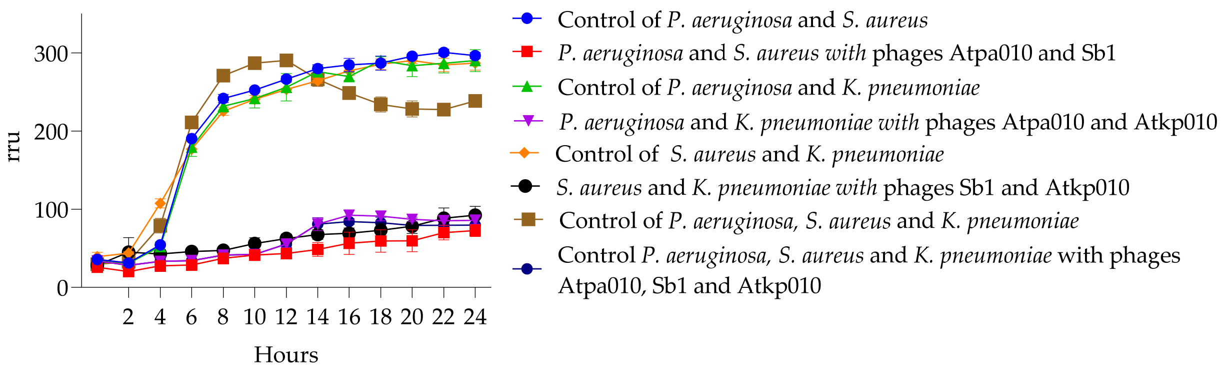

(b)

Figure S2. Antibiotic sensitivity test validated by the VITEK 2 and performed at the QAMH Military Medical Laboratory Capacity (MMLC). The original strains of (a) *P. aeruginosa* PAO1K, (b) *S. aureus* ATCC3865 and (c) *K. pneumoniae* ATCC27736.

(a)

Militair Hospitaal Koningin Astrid

DEFENSIE - Laboratorium Bruynstraat 1 - 1120 Brussel - Erkeningsnr:82675177-998
Fax : 02/443.39.104 - Tel Srt : 02/443.20.85 - mil: 9-6321- 32085 - Mail: MHKA-MMLC@mil.be

Pa PA01K

Geboren op (?)

Afname 27/01/2026 08:58 Ontvangst 27/01/2026 08:58 **Volledig Protocol**

Onze Ref : 260127119 Hospi : 28/01/2026
Uw Ref : Pag 1 van 2

Analyses	Resultaten	Ref waarden	Eenheden

BACTERIOLOGIE

Staal GELOSE
Cultuur aeroob 1: Pseudomonas aeruginosa: zeer veel
Antibiogramme

1. Pseudomonas aeruginosa
bacteriologische identificatie - Sample: 2601270429

Ampicilline	(R)
Tetracycline	(R)
Amoxicilline+clavulanique	(R)
Imipenem	2 (H)
Imipenem-Relebactam	0.5 (S)
Ceftazidime	2 (H)
Aztreonam	4 (H)
Amikacine (other)	2 (S)
Ciprofloxacine	0.25 (H)
Cefepime	2 (H)
Meropenem (meningitis)	2 (S)
Meropenem (andere)	2 (S)
Ertapenem	(R)
Kanamycine	(R)
Tobramycine	<= 1 (S)
Chloramphenicol	(R)
Cefotaxime	(R)
Tigecycline	(R)
Piperacillin-Tazobactam	8 (H)
Levofloxacin	0.5 (H)
Ceftolozan-Tazobactam	0.5 (S)
Ceftazidime/Avibactam	2 (S)

VOORLOPIG PROTOCOL

(b)

Militair Hospitaal Koningin Astrid

DEFENSIE - Laboratorium Bruynstraat 1 - 1120 Brussel - Erkeningsnr:82675177-998
Fax : 02/443.39.104 - Tel Srt : 02/443.20.85 - mil: 9-6321- 32085 - Mail: MHKA-MMLC@mil.be

SA 6538

Geboren op (?)

Afname 27/01/2026 08:58 *Ontvangst 27/01/2026 08:58* ***Volledig Protocol***

Onze Ref : 260127118 *Hospi :* *30/01/2026*
Uw Ref : *Pag 1 van 1*

Analyses	Resultaten	Ref waarden	Eenheden
**BACTERIOLOGIE**			
**Staal GELOSE**			
Cultuur aeroob	1: Staphylococcus aureus: zeer veel		
**Antibiogramme**			

1. Staphylococcus aureus
bacteriologische identificatie - Sample: 2601270427

Oxacillin	<= 0.25 (S)
Tetracycline	<= 1 (S)
Amoxicilline+clavulanique	(S)
Minocycline	<= 0.5 (S)
Erythromycine	<= 0.25 (S)
Vancomyine	<= 0.5 (S) ✓
Ciprofloxacine	<= 0.5 (H)
Trimetoprime+Sulfamethoxazole	<= 10 (S)
Mupirocin	<= 1 (S)
Tobramycine	<= 1 (S)
Gentamicine	<= 0.5 (S)
Rifampicine	<= 0.03 (S)
Chloramphenicol	<= 4 (S)
Clindamycine	0.25 (S)
Fusidic Acid	<= 0.5 (S)
Linezolid	1 (S)

Pha. Y. Van der Beken - Pha. A. Stoefs - Dr. A. Vanhonsebrouck - Dr. E. Huyghe

Bij klachten kan u steeds MMLC-MHKA@mil.be contacteren.

VOORLOPIG PROTOCOL

(c)

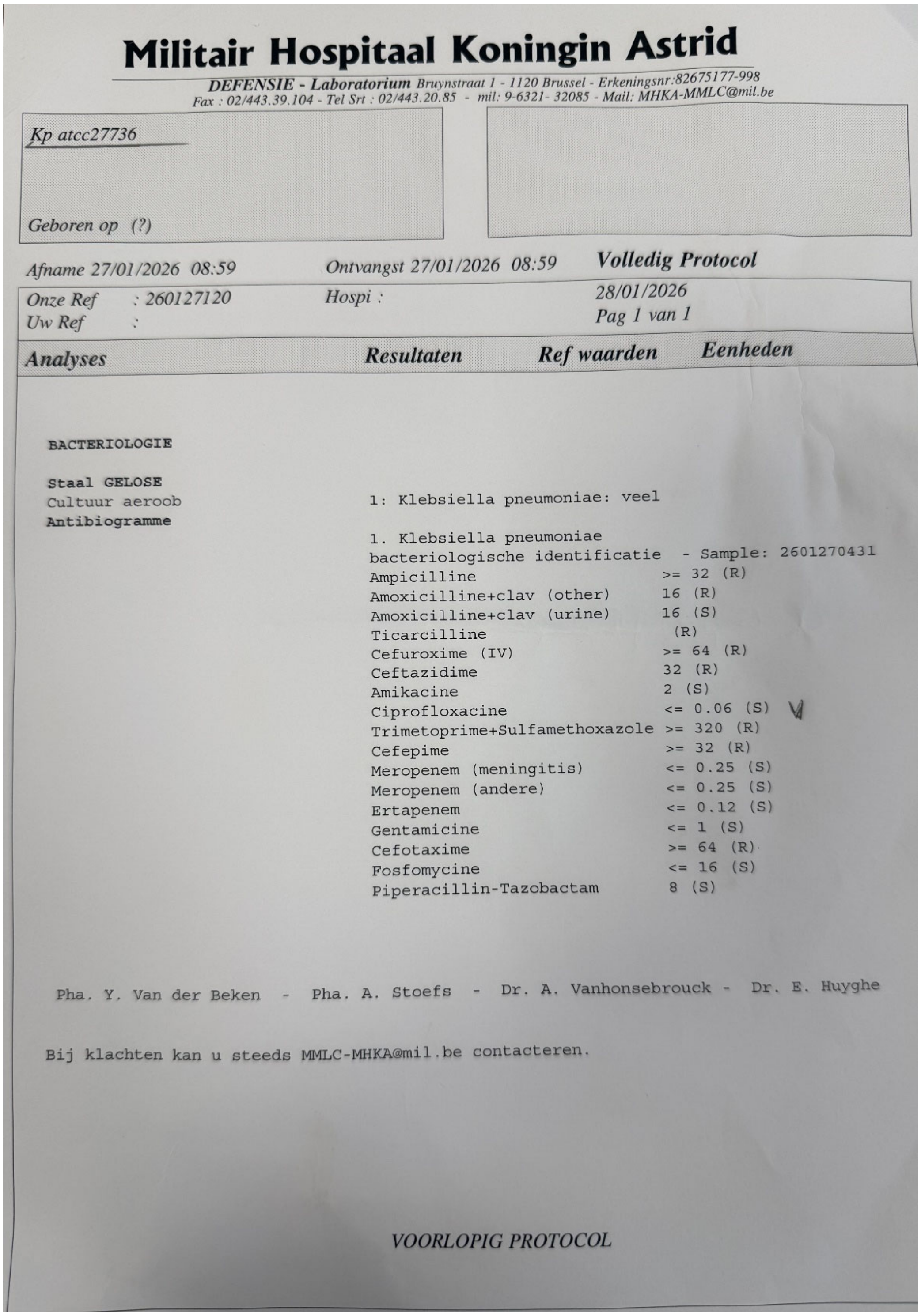

Militair Hospitaal Koningin Astrid

DEFENSIE - Laboratorium Bruynstraat 1 - 1120 Brussel - Erkeningsnr:82675177-998
Fax : 02/443.39.104 - Tel Srt : 02/443.20.85 - mil: 9-6321- 32085 - Mail: MHKA-MMLC@mil.be

Kp atcc27736

Geboren op (?)

Afname 27/01/2026 08:59 *Ontvangst 27/01/2026 08:59* ***Volledig Protocol***

Onze Ref : 260127120 *Hospi :* *28/01/2026*
Uw Ref : *Pag 1 van 1*

Analyses	***Resultaten***	***Ref waarden***	***Eenheden***

BACTERIOLOGIE

Staal GELOSE
Cultuur aeroob — 1: Klebsiella pneumoniae: veel
Antibiogramme

1. Klebsiella pneumoniae
bacteriologische identificatie - Sample: 2601270431

Ampicilline	>= 32 (R)
Amoxicilline+clav (other)	16 (R)
Amoxicilline+clav (urine)	16 (S)
Ticarcilline	(R)
Cefuroxime (IV)	>= 64 (R)
Ceftazidime	32 (R)
Amikacine	2 (S)
Ciprofloxacine	<= 0.06 (S)
Trimetoprime+Sulfamethoxazole	>= 320 (R)
Cefepime	>= 32 (R)
Meropenem (meningitis)	<= 0.25 (S)
Meropenem (andere)	<= 0.25 (S)
Ertapenem	<= 0.12 (S)
Gentamicine	<= 1 (S)
Cefotaxime	>= 64 (R)
Fosfomycine	<= 16 (S)
Piperacillin-Tazobactam	8 (S)

Pha. Y. Van der Beken - Pha. A. Stoefs - Dr. A. Vanhonsebrouck - Dr. E. Huyghe

Bij klachten kan u steeds MMLC-MHKA@mil.be contacteren.

VOORLOPIG PROTOCOL

Figure S3. The proliferation curves of the high concentration planktonic cultures of (a) *P. aeruginosa*, (b) *S. aureus*, (c) *K. pneumoniae*, (d) *P. aeruginosa* and *S. aureus*, (e) *P. aeruginosa* and *K. pneumoniae*, (f) *S. aureus* and *K. pneumoniae*, and (g) *P. aeruginosa*, *S. aureus* and *K. pneumoniae*, exposed to different concentrations of ciprofloxacin. Values are the mean ± SD of triplicates (n = 3).

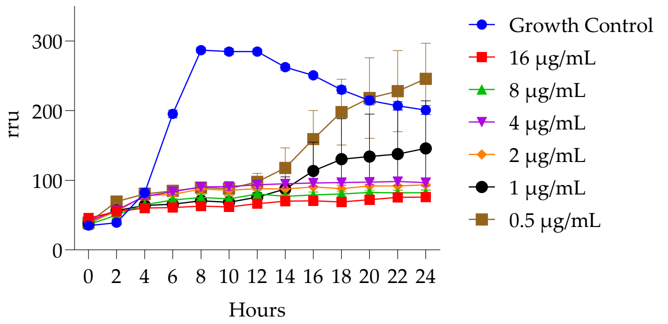

(a)

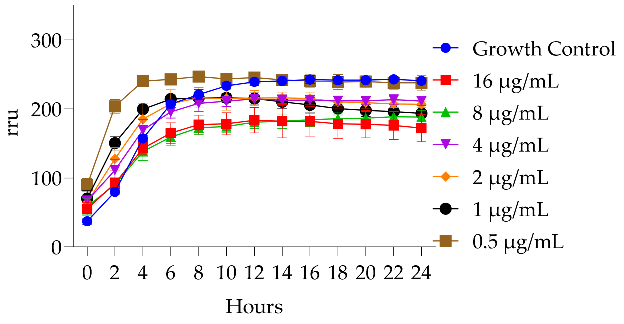

(b)

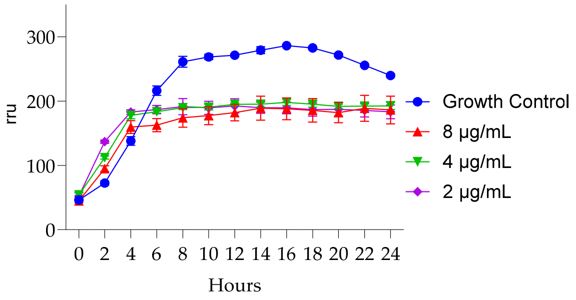

(c)

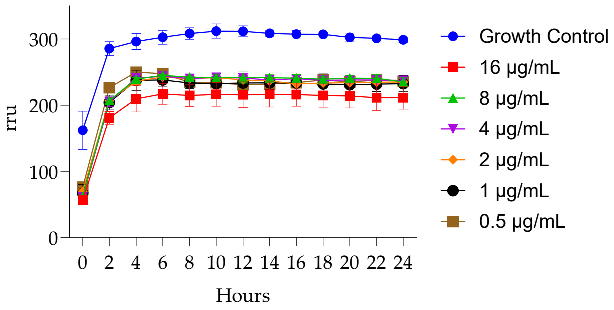

(d)

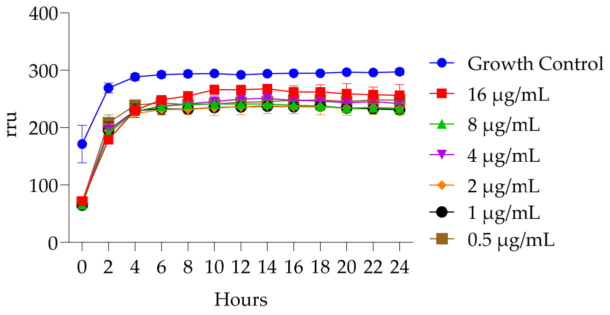

(e)

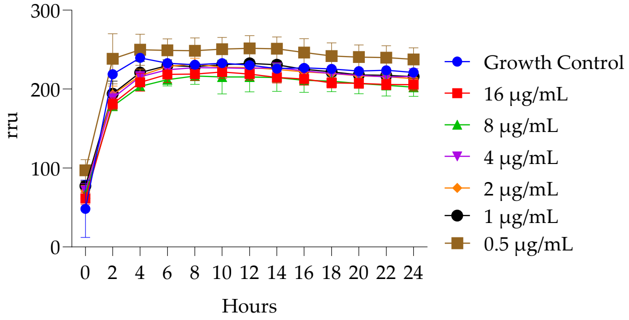

(f)

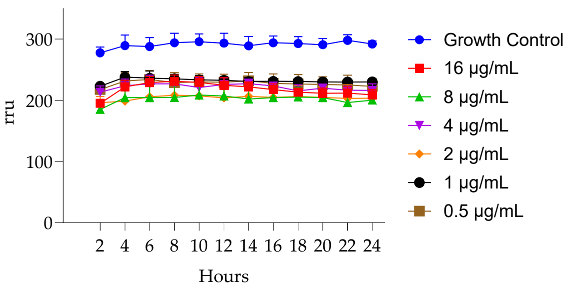

(g)

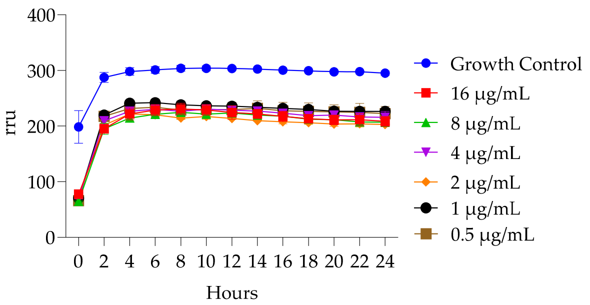

Table S1. Reduction of High-Load Planktonic Cultures and Co-Cultures upon Exposure to 1–8 μg/L of Ciprofloxacin. Values are the mean ± SD of triplicates (n = 3).

Planktonic Culture and Co-culture	Control (RRU)	After treatment (RRU)	Percentage of reduction
S. aureus	264	196	26%
K. pneumoniae	265	192	28%
P. aeruginosa and *S. aureus*	311	219	30%
P. aeruginosa and *K. pneumoniae*	275	231	16%
S. aureus and *K. pneumoniae*	250	205	18%
P. aeruginosa, *S. aureus* and *K. pneumoniae*	292	222	24%

Figure S4. Lytic activity of phages and phage cocktails in high-load planktonic cultures: (a). *P. aeruginosa* phage Atpa010, *S. aureus* phage Sb1, and *K. pneumoniae* phage Atkp010. (b) Phage di-cocktails: Atpa010 and Sb1; Atpa010 and Atkp010; Sb1 and Atkp010; and tri-cocktail: Atpa010, Sb1, and Atkp010. Values are the mean ± SD of triplicates (n = 3).

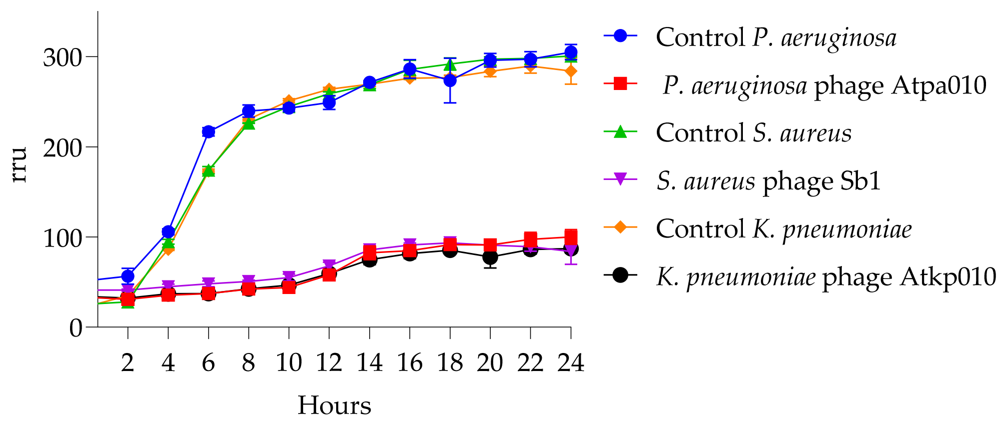

(a)

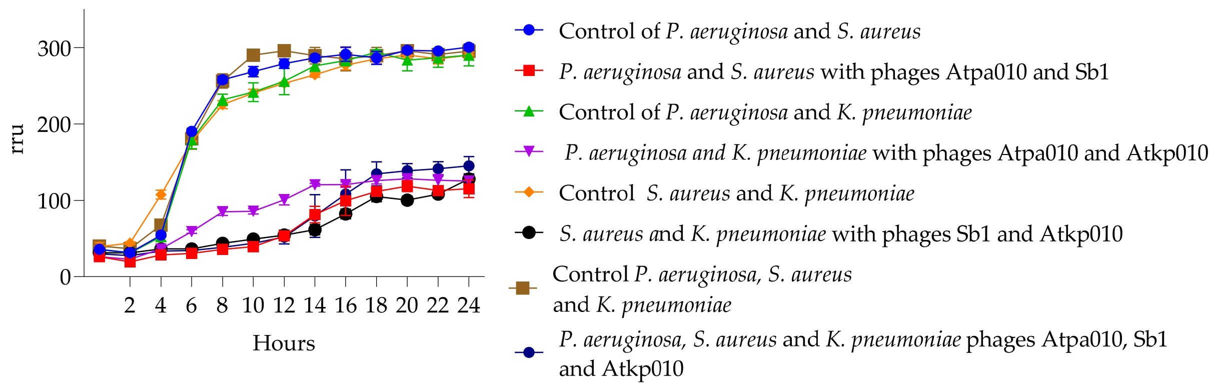

(b)

Figure S5. The antibiotic sensitivity re-test (1), performed using the VITEK 2 at the QAMH Military Medical Laboratory Capacity (MMLC). The biofilm associated isolates of (a) *P. aeruginosa* PAO1K, (b) *S. aureus* ATCC3865 and (c) *K. pneumoniae* ATCC27736.

(a)

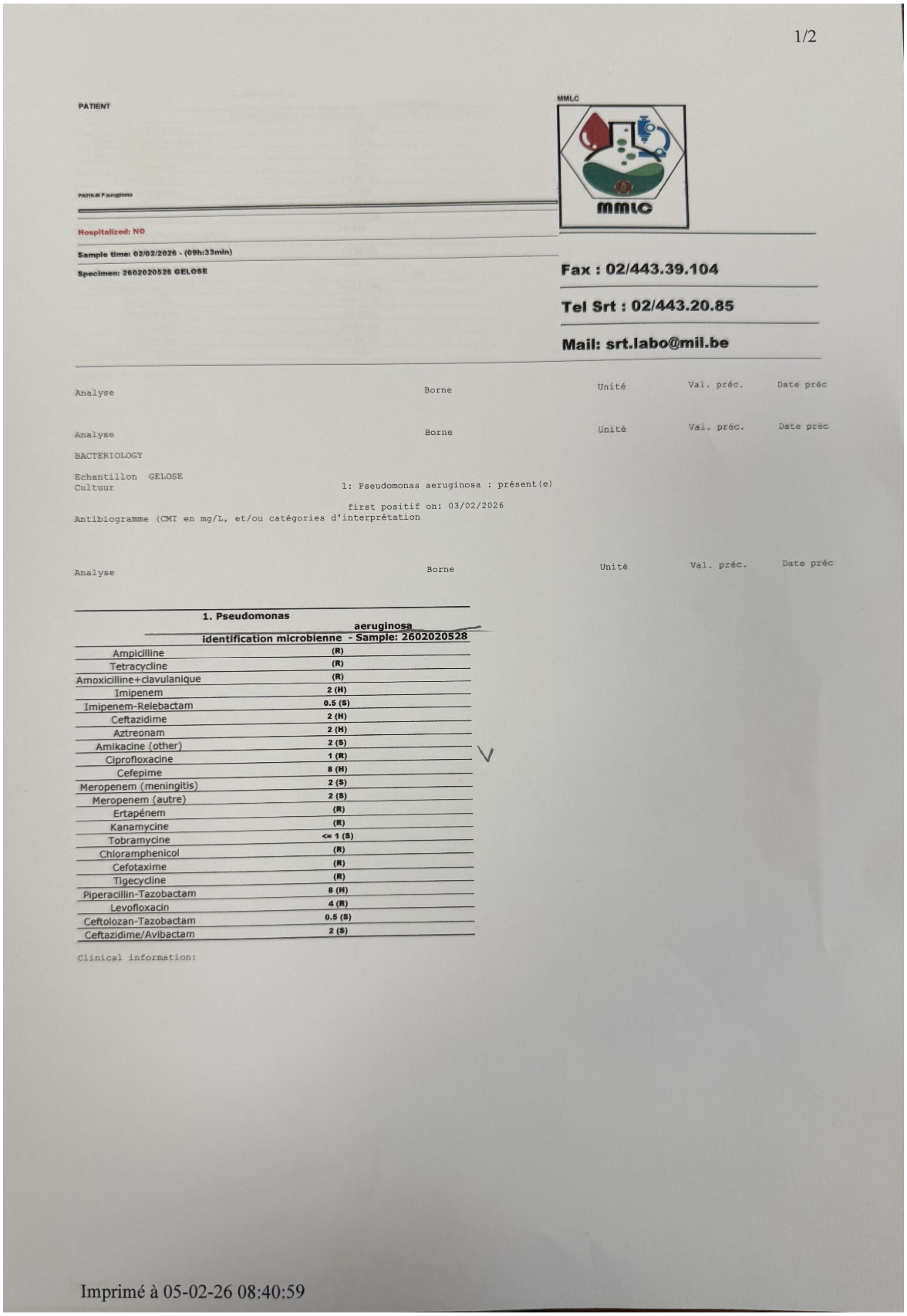

1/2

PATIENT

MMLC

PA01K-8I P.aeruginosa

Hospitalized: NO

Sample time: 02/02/2026 - (09h:33min)

Specimen: 2602020528 GELOSE

Fax : 02/443.39.104

Tel Srt : 02/443.20.85

Mail: srt.labo@mil.be

Analyse	Borne	Unité	Val. préc.	Date préc

Analyse	Borne	Unité	Val. préc.	Date préc

BACTERIOLOGY

Echantillon GELOSE

Cultuur 1: Pseudomonas aeruginosa : présent(e)

first positif on: 03/02/2026

Antibiogramme (CMI en mg/L, et/ou catégories d'interprétation

Analyse	Borne	Unité	Val. préc.	Date préc

1. Pseudomonas aeruginosa identification microbienne	- Sample: 2602020528
Ampicilline	(R)
Tetracycline	(R)
Amoxicilline+clavulanique	(R)
Imipenem	2 (H)
Imipenem-Relebactam	0.5 (S)
Ceftazidime	2 (H)
Aztreonam	2 (H)
Amikacine (other)	2 (S)
Ciprofloxacine	1 (R)
Cefepime	8 (H)
Meropenem (meningitis)	2 (S)
Meropenem (autre)	2 (S)
Ertapénem	(R)
Kanamycine	(R)
Tobramycine	<= 1 (S)
Chloramphenicol	(R)
Cefotaxime	(R)
Tigecycline	(R)
Piperacillin-Tazobactam	8 (H)
Levofloxacin	4 (R)
Ceftolozan-Tazobactam	0.5 (S)
Ceftazidime/Avibactam	2 (S)

Clinical information:

Imprimé à 05-02-26 08:40:59

(b)

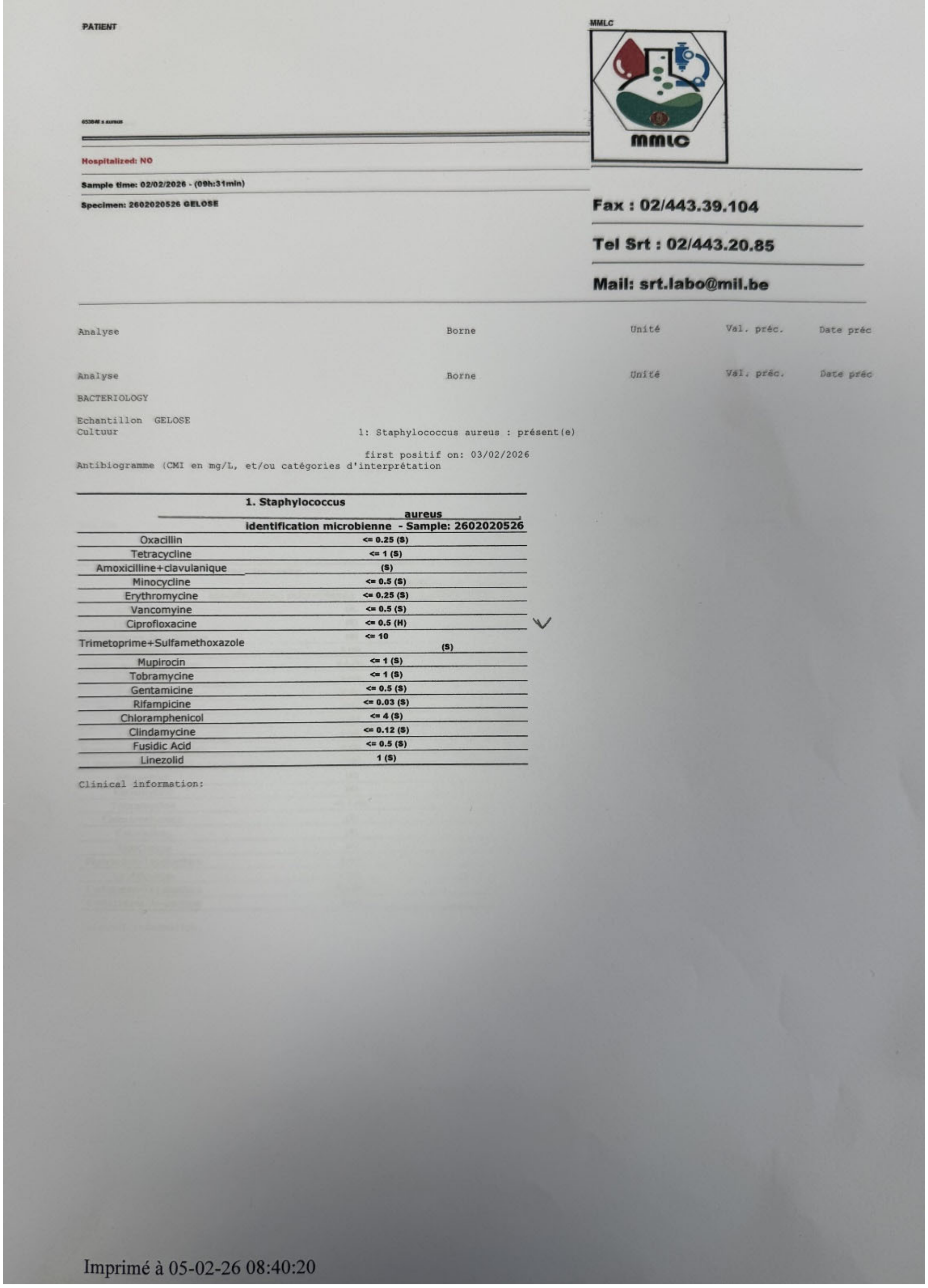

PATIENT

[illegible]

Hospitalized: NO

Sample time: 02/02/2026 - (09h:31min)

Specimen: 2602020526 GELOSE

MMLC

Fax : 02/443.39.104

Tel Srt : 02/443.20.85

Mail: srt.labo@mil.be

Analyse Borne Unité Val. préc. Date préc

Analyse Borne Unité Val. préc. Date préc

BACTERIOLOGY

Echantillon GELOSE
Cultuur 1: Staphylococcus aureus : présent(e)

first positif on: 03/02/2026

Antibiogramme (CMI en mg/L, et/ou catégories d'interprétation

	1. Staphylococcus aureus identification microbienne - Sample: 2602020526
Oxacillin	<= 0.25 (S)
Tetracycline	<= 1 (S)
Amoxicilline+clavulanique	(S)
Minocycline	<= 0.5 (S)
Erythromycine	<= 0.25 (S)
Vancomyine	<= 0.5 (S)
Ciprofloxacine	<= 0.5 (H)
Trimetoprime+Sulfamethoxazole	<= 10 (S)
Mupirocin	<= 1 (S)
Tobramycine	<= 1 (S)
Gentamicine	<= 0.5 (S)
Rifampicine	<= 0.03 (S)
Chloramphenicol	<= 4 (S)
Clindamycine	<= 0.12 (S)
Fusidic Acid	<= 0.5 (S)
Linezolid	1 (S)

Clinical information:

Imprimé à 05-02-26 08:40:20

(C)

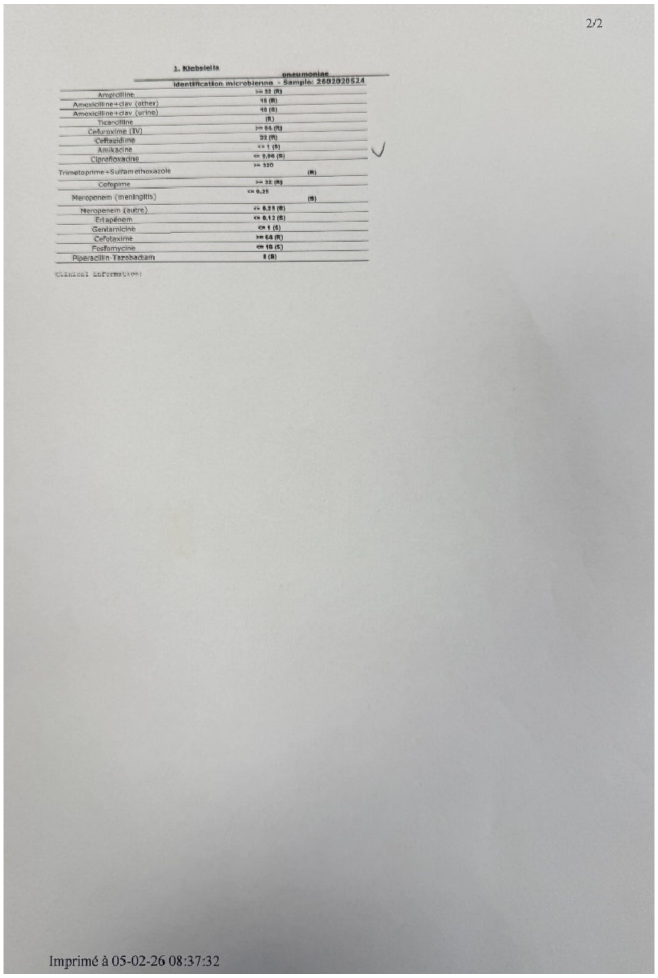

2/2

1. Klebsiella pneumoniae

	Identification microbienne - Sample: 2602020524
Ampicilline	>= 32 (R)
Amoxicilline+clav (other)	16 (R)
Amoxicilline+clav (urine)	16 (S)
Ticarcilline	(R)
Cefuroxime (IV)	>= 64 (R)
Ceftazidime	32 (R)
Amikacine	<= 1 (S)
Ciprofloxacine	<= 0,06 (S)
Trimetoprime+Sulfamethoxazole	>= 320 (R)
Cefepime	>= 32 (R)
Meropenem (meningitis)	<= 0.25 (S)
Meropenem (autre)	<= 0.25 (S)
Ertapénem	<= 0.12 (S)
Gentamicine	<= 1 (S)
Cefotaxime	>= 64 (R)
Fosfomycine	<= 16 (S)
Piperacillin-Tazobactam	8 (S)

Clinical information:

Imprimé à 05-02-26 08:37:32

Figure S6. The antibiotic sensitivity re-test (2), performed using the VITEK 2 at the QAMH Military Medical Laboratory Capacity (MMLC). The biofilm associated isolates of (a) *P. aeruginosa* PAO1K, (b) *S. aureus* ATCC3865 and (c) *K. pneumoniae* ATCC27736.

(a)

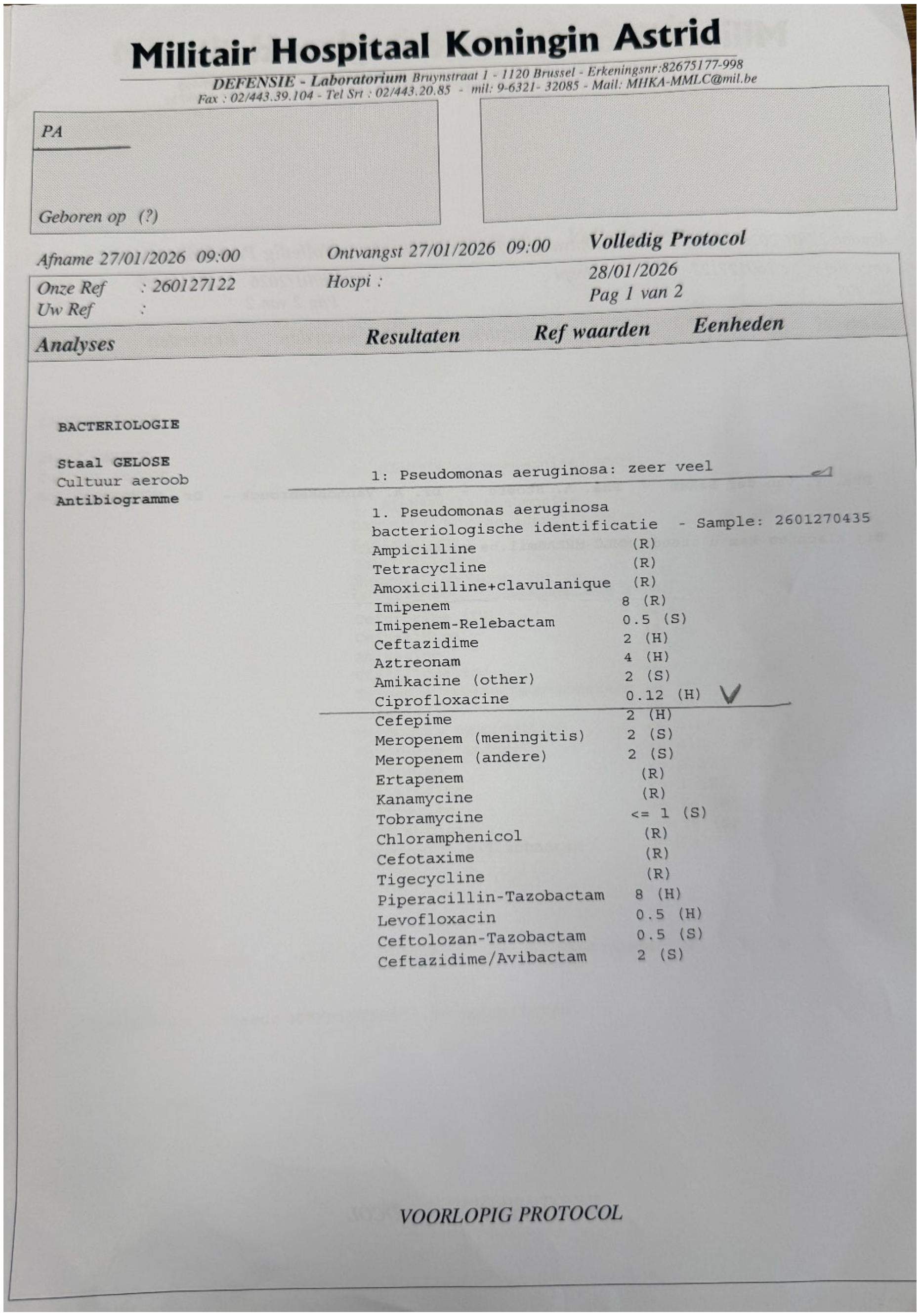

Militair Hospitaal Koningin Astrid

DEFENSIE - Laboratorium Bruynstraat 1 - 1120 Brussel - Erkenningsnr:82675177-998
Fax : 02/443.39.104 - Tel Srt : 02/443.20.85 - mil: 9-6321- 32085 - Mail: MHKA-MMLC@mil.be

PA

Geboren op (?)

Afname 27/01/2026 09:00 *Ontvangst 27/01/2026 09:00* ***Volledig Protocol***

Onze Ref : 260127122 *Hospi :* *28/01/2026*
Uw Ref : *Pag 1 van 2*

Analyses	***Resultaten***	***Ref waarden***	***Eenheden***
**BACTERIOLOGIE**			
**Staal GELOSE**			
Cultuur aeroob	1: Pseudomonas aeruginosa: zeer veel		
**Antibiogramme**			

1. Pseudomonas aeruginosa
bacteriologische identificatie - Sample: 2601270435

Ampicilline	(R)
Tetracycline	(R)
Amoxicilline+clavulanique	(R)
Imipenem	8 (R)
Imipenem-Relebactam	0.5 (S)
Ceftazidime	2 (H)
Aztreonam	4 (H)
Amikacine (other)	2 (S)
Ciprofloxacine	0.12 (H)
Cefepime	2 (H)
Meropenem (meningitis)	2 (S)
Meropenem (andere)	2 (S)
Ertapenem	(R)
Kanamycine	(R)
Tobramycine	<= 1 (S)
Chloramphenicol	(R)
Cefotaxime	(R)
Tigecycline	(R)
Piperacillin-Tazobactam	8 (H)
Levofloxacin	0.5 (H)
Ceftolozan-Tazobactam	0.5 (S)
Ceftazidime/Avibactam	2 (S)

VOORLOPIG PROTOCOL

(b)

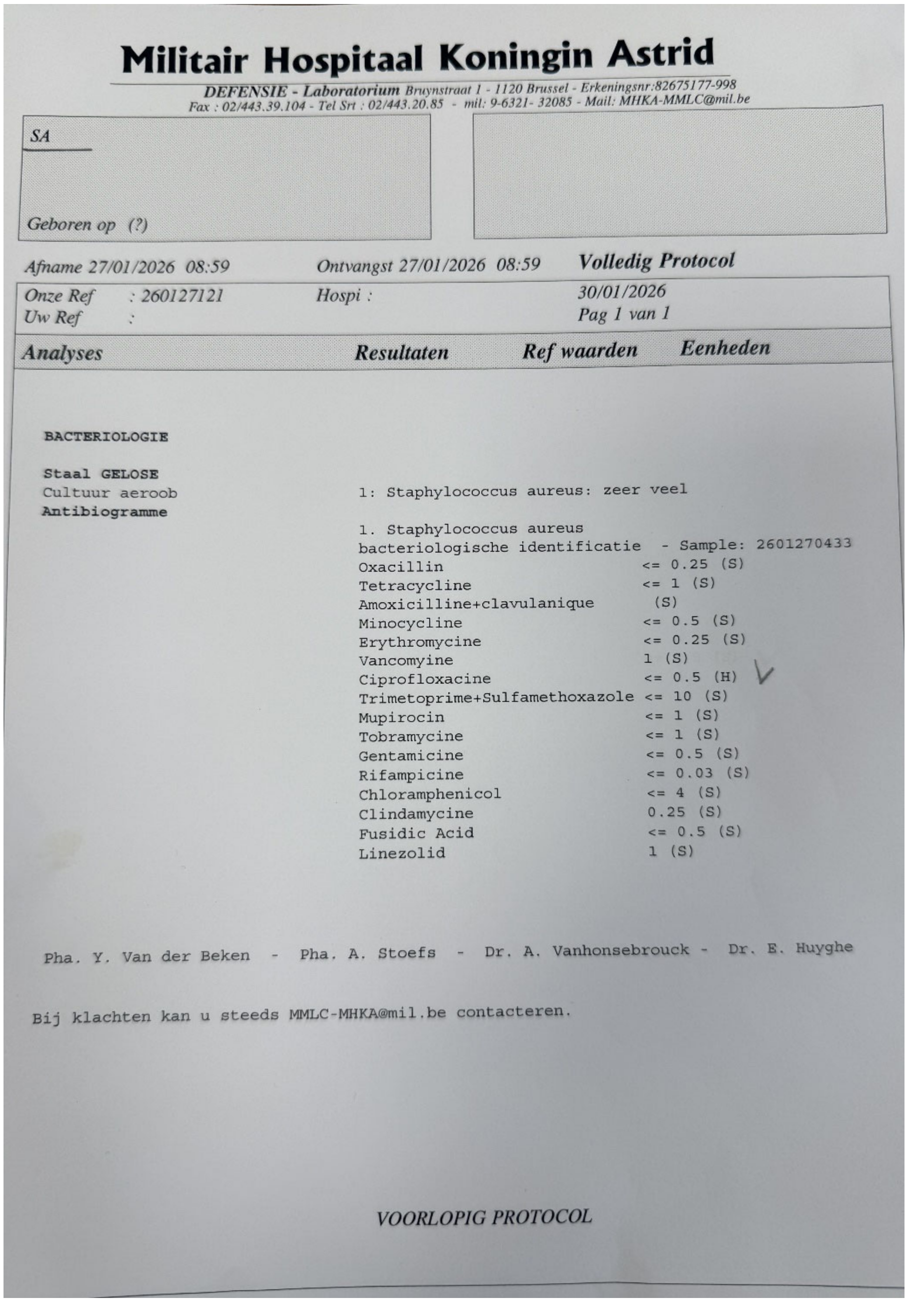

Militair Hospitaal Koningin Astrid

DEFENSIE - Laboratorium Bruynstraat 1 - 1120 Brussel - Erkeningsnr:82675177-998
Fax : 02/443.39.104 - Tel Srt : 02/443.20.85 - mil: 9-6321- 32085 - Mail: MHKA-MMLC@mil.be

SA

Geboren op (?)

Afname 27/01/2026 08:59 *Ontvangst 27/01/2026 08:59* ***Volledig Protocol***

Onze Ref : 260127121 *Hospi :* *30/01/2026*
Uw Ref : *Pag 1 van 1*

Analyses	***Resultaten***	***Ref waarden***	***Eenheden***

BACTERIOLOGIE

**Staal GELOSE**		
Cultuur aeroob	1: Staphylococcus aureus: zeer veel	
**Antibiogramme**	1. Staphylococcus aureus	
	bacteriologische identificatie - Sample: 2601270433	
	Oxacillin	<= 0.25 (S)
	Tetracycline	<= 1 (S)
	Amoxicilline+clavulanique	(S)
	Minocycline	<= 0.5 (S)
	Erythromycine	<= 0.25 (S)
	Vancomyine	1 (S)
	Ciprofloxacine	<= 0.5 (H) ✓
	Trimetoprime+Sulfamethoxazole	<= 10 (S)
	Mupirocin	<= 1 (S)
	Tobramycine	<= 1 (S)
	Gentamicine	<= 0.5 (S)
	Rifampicine	<= 0.03 (S)
	Chloramphenicol	<= 4 (S)
	Clindamycine	0.25 (S)
	Fusidic Acid	<= 0.5 (S)
	Linezolid	1 (S)

Pha. Y. Van der Beken - Pha. A. Stoefs - Dr. A. Vanhonsebrouck - Dr. E. Huyghe

Bij klachten kan u steeds MMLC-MHKA@mil.be contacteren.

VOORLOPIG PROTOCOL

(c)

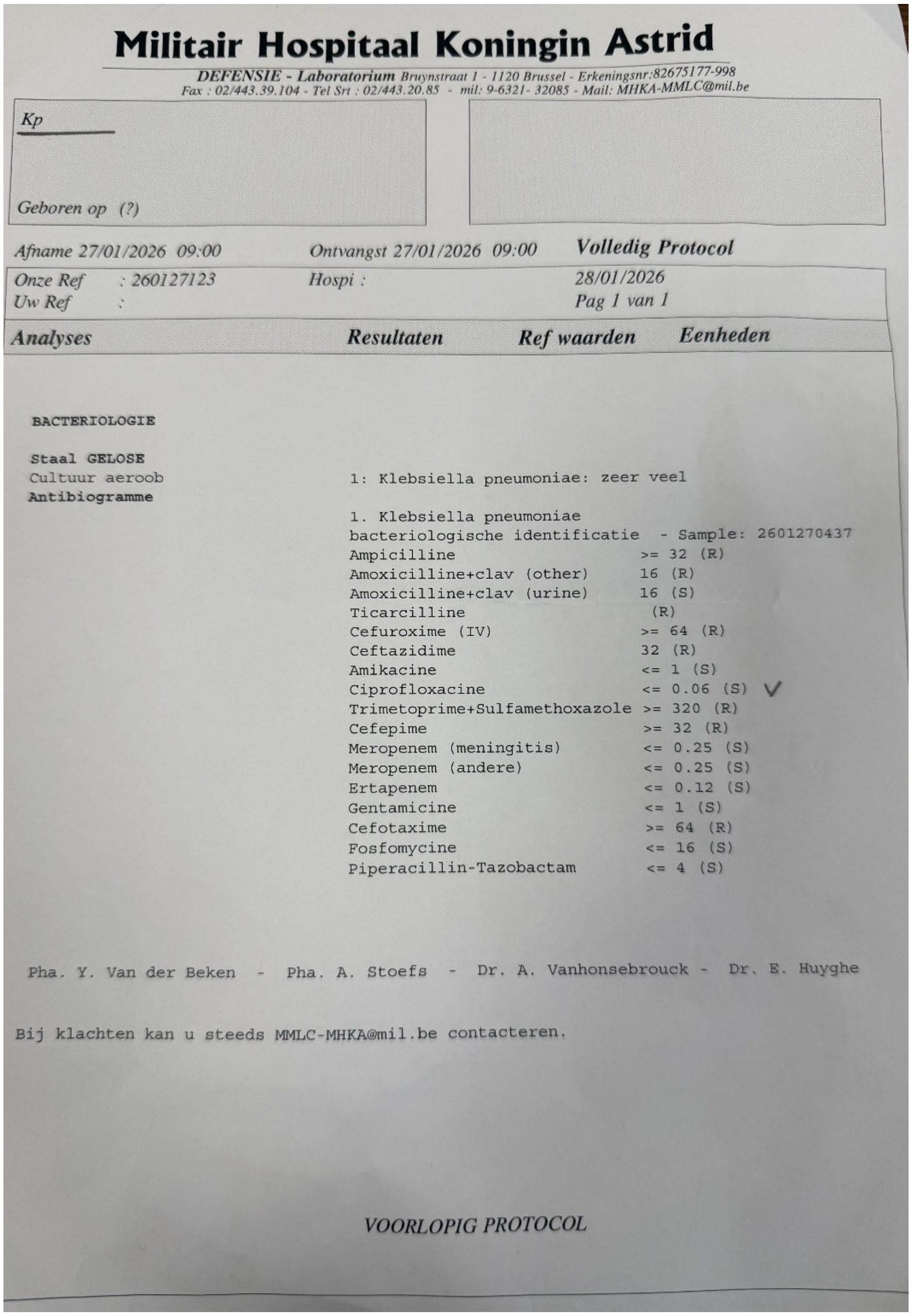

Militair Hospitaal Koningin Astrid

DEFENSIE - Laboratorium Bruynstraat 1 - 1120 Brussel - Erkenningsnr:82675177-998
Fax : 02/443.39.104 - Tel Srt : 02/443.20.85 - mil: 9-6321- 32085 - Mail: MHKA-MMLC@mil.be

Kp

Geboren op (?)

Afname 27/01/2026 09:00 — Ontvangst 27/01/2026 09:00 — **Volledig Protocol**

Onze Ref : 260127123 — Hospi : — 28/01/2026
Uw Ref : — Pag 1 van 1

Analyses	Resultaten	Ref waarden	Eenheden

BACTERIOLOGIE

Staal GELOSE

Cultuur aeroob	1: Klebsiella pneumoniae: zeer veel	
**Antibiogramme**	1. Klebsiella pneumoniae	
	bacteriologische identificatie - Sample: 2601270437	
	Ampicilline	>= 32 (R)
	Amoxicilline+clav (other)	16 (R)
	Amoxicilline+clav (urine)	16 (S)
	Ticarcilline	(R)
	Cefuroxime (IV)	>= 64 (R)
	Ceftazidime	32 (R)
	Amikacine	<= 1 (S)
	Ciprofloxacine	<= 0.06 (S) ✓
	Trimetoprime+Sulfamethoxazole	>= 320 (R)
	Cefepime	>= 32 (R)
	Meropenem (meningitis)	<= 0.25 (S)
	Meropenem (andere)	<= 0.25 (S)
	Ertapenem	<= 0.12 (S)
	Gentamicine	<= 1 (S)
	Cefotaxime	>= 64 (R)
	Fosfomycine	<= 16 (S)
	Piperacillin-Tazobactam	<= 4 (S)

Pha. Y. Van der Beken - Pha. A. Stoefs - Dr. A. Vanhonsebrouck - Dr. E. Huyghe

Bij klachten kan u steeds MMLC-MHKA@mil.be contacteren.

VOORLOPIG PROTOCOL

Figure S7. Pictures of enumeration plates. (a) The *S. aureus* isolates from the dual-species biofilm of *P. aeruginosa* and *S. aureus* appeared after 48–72 hours of culturing on an agar plate. (b) The *S. aureus* isolates from the dual-species biofilm of *S. aureus* and *K. pneumoniae* appeared after 48-72 hours of culturing on an agar plate. (c) The *P. aeruginosa* isolates from the dual-species biofilm of *P. aeruginosa* and *K. pneumoniae* appeared after 48 hours of

culturing on an agar plate. (d) and (e) The *P. aeruginosa* isolates from the ciprofloxacin-treated dual-species biofilm of *P. aeruginosa* and *S. aureus* appeared after 48 hours of culturing on an agar plate. (f) The *P. aeruginosa* isolate from the ciprofloxacin-treated poly-species biofilm appeared after 48 hours of culturing on an agar plate. (g) Normal-sized and small colony variants of biofilm isolate of *K. pneumoniae.*

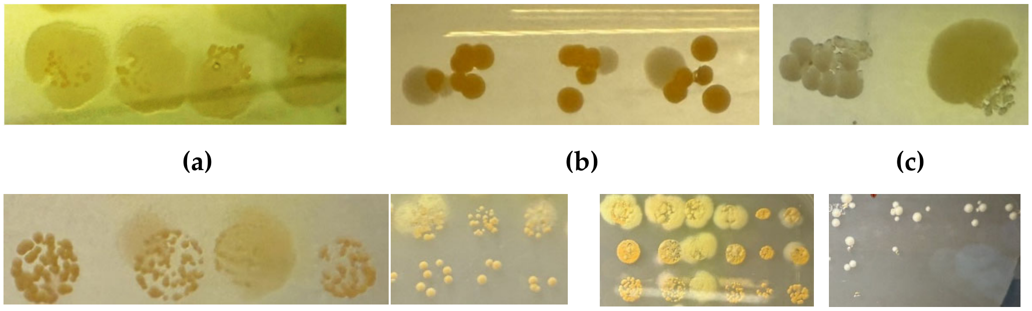

(a) (b) (c)

(d) (e) (f) (g)
